# A 3D printable tissue adhesive

**DOI:** 10.1038/s41467-024-45147-9

**Published:** 2024-02-09

**Authors:** Sarah J. Wu, Jingjing Wu, Samuel J. Kaser, Heejung Roh, Ruth D. Shiferaw, Hyunwoo Yuk, Xuanhe Zhao

**Affiliations:** 1 Department of Mechanical Engineering, Massachusetts Institute of Technology, Cambridge, MA 02139 USA; 2 Department of Chemistry, Massachusetts Institute of Technology, Cambridge, MA 02139 USA; 3 Department of Civil and Environmental Engineering, Massachusetts Institute of Technology, Cambridge, MA 02139 USA; 4 Present Address: SanaHeal, Inc., Cambridge, MA USA

**Keywords:** Polymers, Biomedical engineering, Biomedical materials, Implants, Wetting

## Abstract

Tissue adhesives are promising alternatives to sutures and staples for joining tissues, sealing defects, and immobilizing devices. However, existing adhesives mostly take the forms of glues or hydrogels, which offer limited versatility. We report a direct-ink-write 3D printable tissue adhesive which can be used to fabricate bioadhesive patches and devices with programmable architectures, unlocking new potential for application-specific designs. The adhesive is conformable and stretchable, achieves robust adhesion with wet tissues within seconds, and exhibits favorable biocompatibility. In vivo rat trachea and colon defect models demonstrate the fluid-tight tissue sealing capability of the printed patches, which maintained adhesion over 4 weeks. Moreover, incorporation of a blood-repelling hydrophobic matrix enables the printed patches to seal actively bleeding tissues. Beyond wound closure, the 3D printable adhesive has broad applicability across various tissue-interfacing devices, highlighted through representative proof-of-concept designs. Together, this platform offers a promising strategy toward developing advanced tissue adhesive technologies.

## Abstract (web summary)

Tissue adhesives have received significant interest for their clinical utility but are typically incompatible with advanced manufacturing methods. Here, the authors introduce a 3D printable tissue adhesive for the fabrication of patches and devices for diverse biomedical applications.

## Introduction

Over 300 million surgical procedures are performed globally each year, and most of these operations require efforts to join tissues, seal wounds, or implant devices^1^. Tissue adhesive biomaterials are attractive tools for performing such tasks, as they offer ease of use and minimal tissue damage compared to traditional sutures and staples^2–10^. Ideally, a tissue adhesive should be able to achieve fast, conformable, strong, and biocompatible adhesion with tissues. Efforts toward achieving these properties have resulted in a range of adhesive materials, among which bioadhesive hydrogels have emerged as promising candidates for realizing tough adhesion and good biocompatibility^9,11–14^. However, typical mold-casted hydrogels provide limited manufacturing freedom over their shapes and properties, constraining their versatility and offering little room for development toward more customized, application-specific technologies.

3D printing has become a forefront technology for manufacturing biomedical products with controlled architectures, proving useful for constructing devices customized for different host tissues or functionalities^15,16^. While there have been significant efforts toward developing 3D printable biomaterials for tissue scaffolds, prosthetics, and pharmaceutics, the potential for 3D printing tissue adhesives has remained largely unexplored^17–19^. A main obstacle lies in the challenging set of material properties required to ensure both printability and high adhesion performance: a printable tissue adhesive must possess proper rheological properties for layer stacking (e.g., shear thinning behavior and sufficient yield stress) while retaining its adhesive properties through the printing and post-processing steps^20^.

**Fig. 1:**
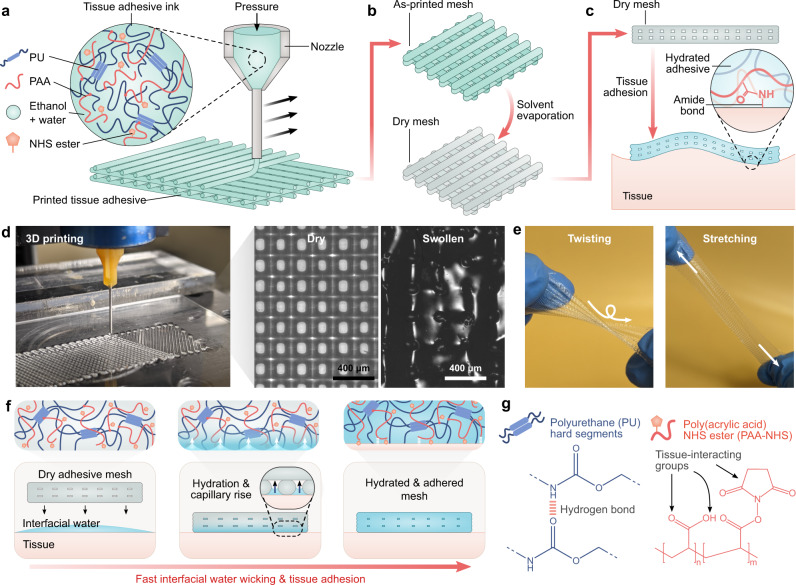
Fabrication and adhesion of the 3D printable tissue adhesive. **a**–**c** Schematic illustration of the 3D printing overview. A tissue adhesive ink is extruded using direct-ink-write 3D printing and dried to obtain an adhesive mesh patch. The mesh is then applied in the dry state to a hydrated tissue surface, where it achieves adhesion by forming covalent amide bonds with primary amines on the tissue surface. **d** Photograph of the 3D printing process and micrographs of a 3D printed mesh patch in dry and swollen states. **e** Photographs of as-printed patches being twisted and stretched. **f** Schematic illustration of the adhesion mechanism. Interfacial water is swiftly removed via hydration of the material and capillary rise through the mesh pores, which enables rapid consolidation with tissues. The formation of intermolecular and covalent bonds stabilizes adhesion. **g** Key chemical moieties of the 3D printable tissue adhesive copolymer: Hydrogen bond-forming groups in polyurethane (PU), and tissue-interacting groups in poly(acrylic acid) (PAA) coupled with NHS ester.

Here, we present a direct-ink-write 3D printable (3DP) tissue adhesive ink that enables the additive manufacturing of structures with tunable geometries and robust tissue adhesive properties (Fig. 1a–c). Specifically, we develop a polymer network comprised of poly(acrylic) acid functionalized with *N*-hydroxysuccinimide ester (PAA-NHS ester) interpenetrated with and grafted to a hydrophilic polyurethane (PU). A key innovation of this material is the ability to process it into a viscoelastic ink by dissolution in a benign solvent (30 v/v % water and 70 v/v % ethanol), making it amenable to extrusion-based 3D printing (Fig. 1d–e). The polymer constituents are selected based on design principles for achieving rapid and robust adhesion: specifically, leveraging hydrophilic moieties to enable rapid adhesion, and incorporating strong interfacial linkages and bulk energy dissipation mechanisms to enhance interfacial toughness^9,11,21^ (Fig. 1f). Owing to a high density of charged carboxylic acid groups, the hydrophilic PAA chains facilitate rapid interfacial water uptake and can quickly consolidate with tissue surfaces to form intermolecular bonds^9,22,23^. Reactive NHS ester groups further contribute to tissue adhesion by interacting with primary amines on tissues to form covalent amide bonds. Meanwhile, the hard segments in PU interact via dynamic hydrogen bonding, providing an energy dissipation mechanism under deformation^21^ (Fig. 1g). Previously, a commercially-available thermoplastic ether-based hydrophilic PU has demonstrated favorable biocompatibility and mechanical properties (i.e., high stretchability and toughness)^9,24–26^. Here, this off-the-shelf material is leveraged as a base polymer in the tissue adhesive to impart these favorable properties to the resulting graft interpenetrating network. The elastomeric mechanical properties of the resulting polymer make it suitable for conforming to varying wound geometries and bearing loads in dynamic tissues (Fig. 1e and Supplementary Fig. 1). We demonstrate how 3D printing of this material creates a streamlined and versatile fabrication platform for designing tissue adhesive structures for tissue repair and bio-integrated devices.

## Results

### 3D printable tissue adhesive ink

**Fig. 2:**
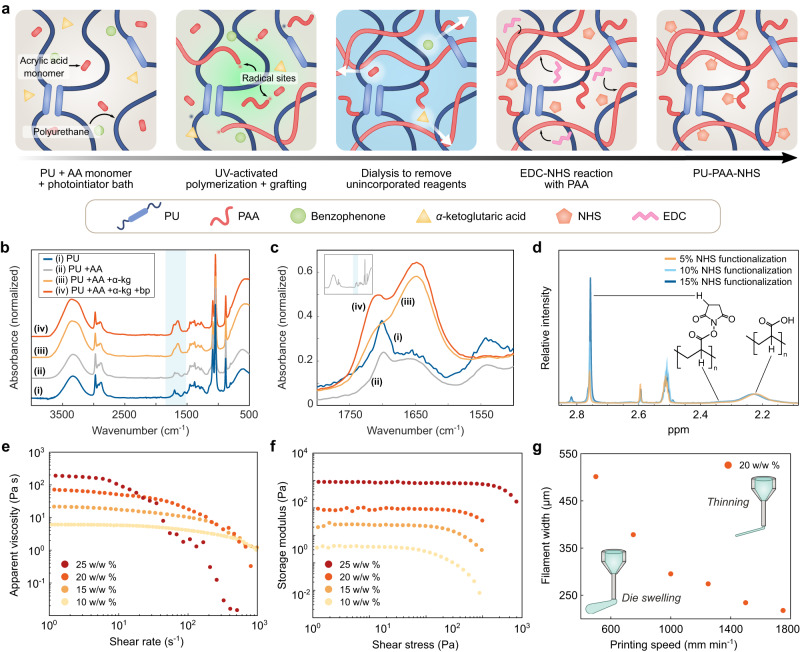
Synthesis and material characterization of the 3D printable tissue adhesive. **a** Illustrated schematic of the tissue adhesive synthesis process. Polyurethane (PU), acrylic acid (AA), and photoinitiators are combined in a precursor solution and exposed to UV light to activate a one-pot nonspecific polymerization reaction, resulting in poly(acrylic acid) (PAA) grafted to and entangled with PU. The unincorporated reagents are removed via dialysis with ethanol followed by water. The purified material is then redissolved and functionalized with *N*-hydroxysuccinimide (NHS) esters using 3-(dimethylamino)propyl)carbodiimide (EDC) as a coupling agent, yielding PU-PAA-NHS. **b**-**c** FTIR spectra for materials prepared using different precursor compositions. Each spectrum was normalized based on the peak at 2900 cm^−1^. **d** ^1^H NMR spectra for tissue adhesive samples with varying degrees of NHS functionalization. Each spectrum was normalized based on the peak around 2.22 ppm (attributed to PAA), and the degree of NHS functionalization was estimated by integrating the NHS alkyl peak around 2.76 ppm with respect to the normalized peak. **e** Apparent viscosity of tissue adhesive inks as a function of shear stress for varying polymer concentrations. **f** Shear storage modulus as a function of shear stress for varying polymer concentrations. **g** Filament width as a function of printing speed for the tissue adhesive ink comprising 15 w/w % PU-PAA and 5 w/w % PU extruded through a 200 µm-diameter nozzle under 250 kPa of pressure. Values represent the mean (*n* = 3 independent samples). Source data are provided as a Source Data file.

The synthesis of the PU-PAA graft interpenetrating network is achieved using a photoinitiated polymerization method (see Methods section for the detailed procedure) (Fig. 2a). Briefly, UV-assisted synthesis is carried out in a one-pot solution containing PU, acrylic acid, *α*-ketoglutaric acid, and benzophenone. *α*-ketoglutaric acid functions as an initiator for the polymerization of acrylic acid, and benzophenone functions as an initiator for producing radical sites in the PU, which can give rise to covalent grafting of PAA to PU^27–30^. Next, the PAA-grafted PU (PU-PAA) product is purified by dialysis in ethanol followed by water to remove unreacted reagents, and then fully dried to obtain a shelf-stable polymer (Supplementary Figs. 2–3). FTIR analysis of the material substantiates the roles of both initiators in the incorporation of PAA, reflected in the peak around 1710 cm^-1^ associated with the C = O stretching in carboxylic acid (Fig. 2b–c and Supplementary Fig. 4)^27^. The content of acrylic acid and PU in the precursor solution was determined based on achieving a large ratio of PAA:PU in the final product. Specifically, a 4:1 AA:PU weight ratio in the precursor solution was found to produce materials within the upper limit of final PAA content (Supplementary Fig. 5). Prior to 3D printing, the PAA-PU solutions may be blended with PU to further tune the PU content of the ink as desired. This method of polymerizing and crosslinking PAA with PU prior to 3D printing enables the facile direct-ink-writing of tissue adhesive inks which require no further post-processing asides from drying. This contrasts with 3D printing a hydrogel precursor requiring post-processing (e.g., UV curing or chemical crosslinking reactions) which increases the complexity of fabrication and removal of residual monomers.

### 3D printing and adhesion performance

**Fig. 3:**
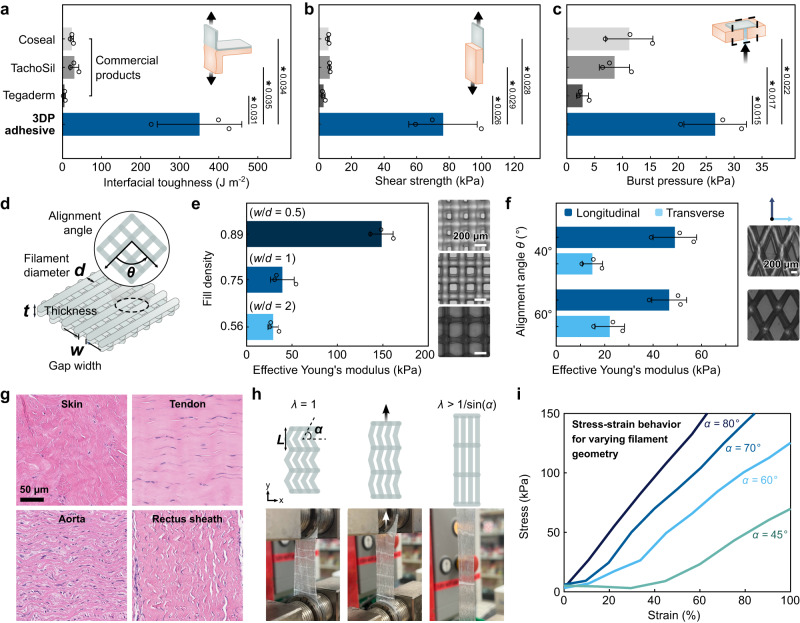
Adhesion performance and mechanical tunability of the 3D printable tissue adhesive. Interfacial toughness (**a**), shear strength (**b**), and burst pressure (**c**) of the 3DP tissue adhesive and commercially available tissue adhesives adhered to wet porcine skin. **d** Tunable geometric parameters of 3D printed structures. **e** Effective initial Young’s moduli for 3D printed patches with varying fill density. **f**, Effective anisotropic Young’s moduli for 3D printed patches with varying alignment angles between alternating printed layers. **g** Histological images stained with H&E of different types of porcine tissues featuring distinct collagen fibril patterns. **h** Illustrated schematic and corresponding photographs of uniaxial stretching for a collagen fiber-inspired patch featuring a 3D printed waveform pattern. For a pattern with bend angle α, the corresponding transition stretch between straightening and bending of the fibers is λ = 1/sin(α). **i** Experimental stress-strain curves for 3D printed patches having different angles α = 80, 70°, 60°, and 45°. The corresponding theoretical transition strains for these angles are around 2%, 6%, 15%, and 41%, respectively. Values and error bars in (**a**–**f**) represent the mean and standard deviation (*n* = 3 independent samples). Statistical significance and *p* values were determined using a two-tailed Student’s t-test with unequal variance: *ns p* > 0.05; * *p* ≤ 0.05; ** *p* ≤ 0.01; *** *p* ≤ 0.001. Source data are provided as a Source Data file.

To convert the synthesized PU-PAA into a printable ink, we first dissolve a high concentration of PU-PAA in an aqueous ethanol solution, yielding a viscous resin (Supplementary Fig. 3b–c). The PU-PAA resin is then mixed with 3-(dimethylamino)propyl)carbodiimide (EDC) and *N*-ethyl-*N*′-(, *N*-hydroxysuccinimide) (NHS) to introduce NHS ester functional groups in the PAA chains, yielding around 10% NHS functionalization of the carboxylic acid groups (Fig. 2d). The direct-ink-write printing method requires that the ink be able to flow through a fine nozzle under pressure, retain its shape after extrusion, and support layer stacking^20^. We found that inks comprising a total polymer concentration below 15 w/w % are prone to spreading, which compromises shape fidelity, whereas inks with polymer concentration exceeding 25 w/w % are difficult to extrude due to clogging in the printing nozzles. Inks within the intermediate range of concentrations (e.g., a composition of 15 w/w % PU-PAA, 5 w/w % PU) exhibit suitable properties for 3D printing (Fig. 2e–g). The filament and pore dimensions can be controlled by tuning the extrusion pressure and printing head speed (Fig. 2g). Following deposition onto a substrate, the printed structures are dried with no further processing required. After adhering to tissues, the printed material equilibrates in wet physiological environments with an equilibrium swelling ratio of around 1.75 (Supplementary Figs. 6–7). The bulk material can achieve high interfacial toughness ( > 300 J m^-2^) and adhesive shear strength ( > 75 kPa) when adhered to porcine skin (Fig. 3a–b and Supplementary Fig. 8a–b).

The 3D printability of the optimized tissue adhesive ink allows direct ink writing of lattice-patterned mesh patches designed for sealing tissue defects. Mesh structures, whose voids enhance flexibility and facilitate mass transport, make rational form factors for a tissue-conforming adhesive. The removal of interfacial water at the adhesive-tissue interface is facilitated by capillary effects and drainage through the pores^31^. To fabricate fluid-tight sealant patches, we printed a mesh tissue adhesive pattern directly onto a thin layer of PU to provide a flexible backing layer. The resulting patch can achieve fluid-tight sealing of 3 mm-diameter defects in ex vivo porcine skin, sustaining burst pressures of over 26 kPa (Fig. 3c and Supplementary Figs. 8c, 9) For reference, this is substantially higher than most physiologically relevant pressures, including hypertensive systolic blood pressure (17-19 kPa) and intrathoracic pressures (6-8 kPa)^32–34^.

### Mechanical tunability

Varying the geometric parameters, such as the filament density or the angle of alignment between filaments, can alter the mechanical properties of the mesh and introduce anisotropic or tunable nonlinear behavior (Fig. 3d–f and Supplementary Figs. 10–11). Considering the range of properties observed in different biological tissues, mechanical tunability may be a useful tool for designing a well-matched tissue-material interface and guiding cell interactions or tissue mechanics (Fig. 3g)^35^. For example, inspired by the wavy collagen fibrils in tissues which give rise to nonlinear stress-strain behavior, we printed patches featuring waveforms that generate tissue-like J-shaped stress-strain responses (Fig. 3h–i). Depending on the arclength of the wave (determined here by the bend angle *α* and period *L*), the printed structures exhibit varying stiffening behavior corresponding to shifts in the transition between straightening of the wavy fibers to stretching-dominated deformation. The experimental J-shaped stress-strain curves plotted in Fig. 3i show different transition points for patterns having different bend angle *α*, which are in close approximation with the theoretical transition stretch of *λ* = 1/sin(*α*), indicating that the 3D printing platform can be leveraged as an avenue for developing patches with programmable nonlinear mechanical properties. In turn, these mechanical properties have the potential to influence the biological response to the implanted material.

### Biocompatibility and degradability

**Fig. 4:**
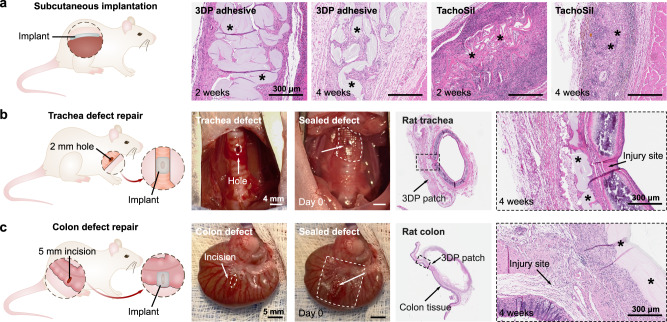
In vivo biocompatibility and sutureless repair of defects in rat models. **a** Representative histological images stained with H&E after 2 and 4 weeks following the subcutaneous implantation of 3D printed patches and TachoSil in rats. **b** Representative photographs and histological images stained with H&E of a rat trachea defect repaired using the 3D printed patch after 4 weeks. **c** Representative photographs and histological images stained with H&E of a rat colon defect repaired using the 3D printed patch after 4 weeks. In the histological images, * represents the implanted material. For each of the animal models presented in (**a**–**c**), three independent experiments were conducted with similar results.

To evaluate the biocompatibility of the 3D printed tissue adhesive, we performed in vitro cell and in vivo animal studies. Quantitative analysis using a LIVE/DEAD assay of mouse fibroblast cells co-cultured with medium (DMEM) soaked with the 3D printed patch reveals high cell viability comparable to a pristine control media group (*P* = 0.41) and other commercially available tissue adhesives (*P* = 0.35 for Tachosil and *P* = 0.71 for Coseal) (Supplementary Fig. 12). We further characterized the in vivo biocompatibility and biodegradability by performing subcutaneous implantations of the 3D printed patch in rats (Fig. 4a). After 2- and 4-weeks post-implantation, the implanted material and adjacent tissues were harvested for histological analysis. The 3D printed patch shows mild inflammation at both time points, with a gradual decrease of the implant volume at 4 weeks due to hydrolytic degradation (Fig. 4a and Supplementary Fig. 13)^36^. Subcutaneous implantation of a commercial product (Tachosil) as a control showed comparable levels of inflammation and relatively faster in vivo degradation.

### In vivo defect repair in rat models

Next, we assessed the applicability of the 3D printed patch for sealing tissue defects, specifically focusing on two scenarios: air-leaking tracheal defects and fluid-leaking colon defects (Fig. 4b, c). Defects in the airway and gastrointestinal (GI) tract can be life-threatening conditions that often require significant patient management or surgical treatment^37,38^. For the trachea, current options for defect repair are limited because sutures apply high tensions susceptible to cartilage tearing and can allow air leaks through gaps between punctures, and flowable sealants may be aspirated into and obstruct the airway. Considering these challenges, the flexible, preformed 3D printed mesh is a favorable form factor for achieving tracheal defect repair. To investigate its tissue sealing potential, we applied the 3D printed patch over 2 mm long x 1 mm wide oval-shaped defects in rat tracheas (Fig. 4b). After 10 s of gentle pressure application, the patch formed a circumferential seal around the defect, achieving air-tight adhesion and restoring air ventilation to the lungs. Histological analysis of the tissues harvested after 4 weeks post-implantation indicates that the defects are repaired by the 3D printed patch with no visible sign of leakages or significant tracheal narrowing (Supplementary Figs. 14 and 15). The patch remains partially degraded at the site of implantation after 4 weeks with mild inflammation, consistent with the subcutaneous implantation study. To evaluate the applicability of the 3D printed patch for repairing GI organs, we used the patch to seal 5-mm incisional defects in rat colons (Fig. 4c). Leakages from GI defects are significant clinical challenges that can result in infection, sepsis, and mortality. As with the trachea, the 3D printed patch readily conforms to the colon surface and provides fluid-tight sealing within 10 s with no signs of bowel leakage following the surgery. After 4 weeks, the partially degraded patch remains adhered at the injury site and the colonic defect is repaired without signs of abscesses (Fig. 4c and Supplementary Fig. 16).

**Fig. 5:**
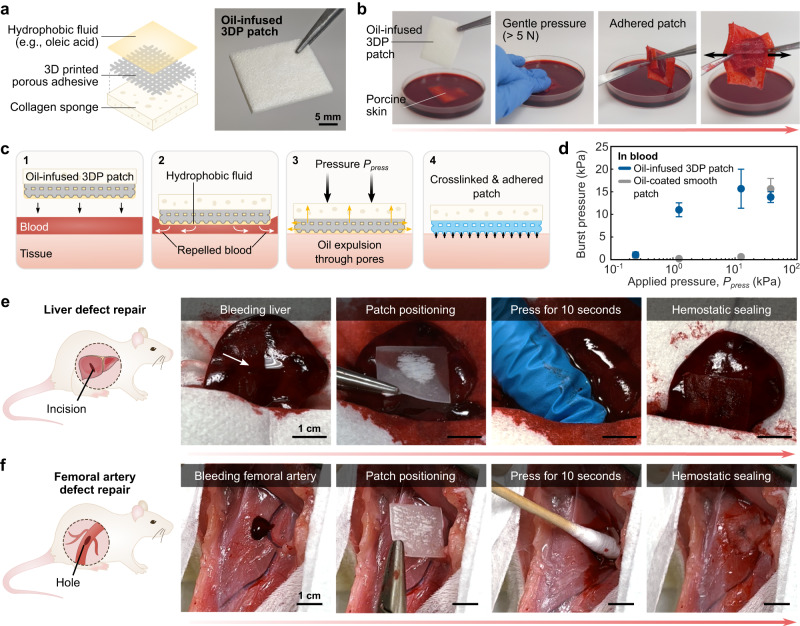
Liquid-infused 3D printed patches for blood-resistant tissue sealing. **a** Illustrated schematic and photograph of the oil-infused 3D printed patch. A porous 3D printed pattern is integrated with a collagen sponge and infiltrated with oleic acid. **b** Photographs of the oil-infused patch being adhered to porcine skin submerged in a blood bath. **c** Illustrated schematic of the blood-repelling, oil-dewetting, and tissue adhesion process. **d** Burst pressure vs. applied pressure for the oil-infused 3D printed patch compared with a smooth, non-porous patch (of the same material) coated with a layer of oil. Compared with the smooth patch, the porous 3D printed patch can achieve higher burst pressures at a lower applied pressure. Values and error bars represent the mean and standard deviation (*n* = 4 independent samples). Source data are provided as a Source Data file. **e** Photographs of the in vivo application of an oil-infused 3D printed patch to repair an actively bleeding defect in the liver of a rat. **f** Photographs of the in vivo application of an oil-infused 3D printed patch to repair an actively bleeding defect in the femoral artery of a rat. For each animal model presented in **e**–**f**, two independent experiments were conducted with similar results.

Addressing severely bleeding injuries is a particularly difficult challenge due to their complex, time-sensitive nature. Many tissue adhesives struggle to adhere in the presence of blood, which can interfere with tissue interactions and wash out liquid adhesives or hemostatic agents. Indeed, the NHS esters which lend adhesive functionality to the PU-PAA-NHS material can be compromised if exposed to blood, limiting the efficacy of the 3D printed patches in extremely bloody scenarios. To address this limitation, we leverage 3D printing to design a liquid-infused patch that resists blood during tissue repair^24,39,40^ (Fig. 5). The liquid-infused patch is based on the incorporation of a hydrophobic (blood-resistant) liquid into a 3D printed porous mesh structure. The efficacy of the liquid-infused patch is underpinned by the favorable thermodynamics governing the interaction between the hydrophobic fluid (i.e., oleic acid) and the textured tissue adhesive surface (Supplementary Fig. 17 and Supplementary Discussion 1). This configuration serves as a physical barrier to preclude fouling by blood and other body fluids, which enables the application of the patch onto actively bleeding defect sites. By exerting adequate pressure, the hydrophobic liquid can be expelled through the mesh’s pores as well as laterally at the interface, allowing the adhesive to form direct tissue contact and subsequently achieve adhesion (Fig. 5a–c). Notably, the pressure requirement for adhering the liquid-infused mesh structure is lower than that for an oil-coated, smooth (non-porous) patch comprised of the same material (Fig. 5d). We hypothesize that This is because in the mesh structure, the hydrophobic fluid may freely drain through the pores, whereas in the smooth patch configuration, it is limited to lateral squeeze-out and may become entrapped at the tissue-patch interface. To further evaluate the blood-resistant adhesive performance, we conducted a series of in vivo experiments to apply the patch to actively bleeding liver and femoral artery defects in live rats (Fig. 5e, f). The liquid-infused 3D printed patch achieves blood-resistant sealing of a bleeding liver injury (a 5 mm-long and 2 mm-deep incision) and a femoral artery injury (a ~ 2 mm snip) within 10 seconds of applied pressure (Supplementary Movies 1-2). In summary, leveraging the 3D printability of the tissue adhesive to create textured, liquid-infused patches demonstrates the additional potential for treating bleeding wounds.

### 3D printing adhesive tissue-interfacing devices

**Fig. 6:**
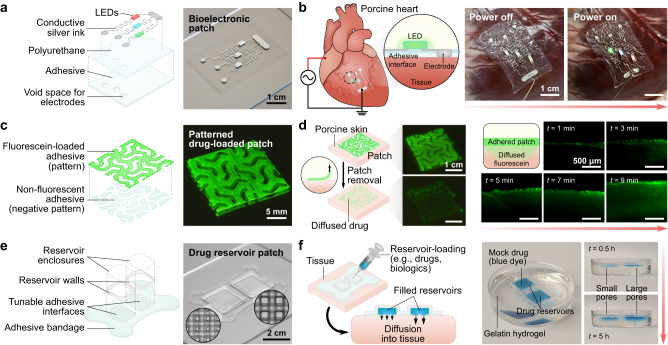
Potential applications of the 3D printable tissue adhesive platform. **a** Design of a 3D printed bioadhesive bioelectronic patch featuring an LED circuit. **b** Ex vivo demonstration of the bioelectronic patch adhered to a porcine heart. Application of a current through the ex vivo heart leads to illumination of the patch-mounted LEDs, demonstrating intimate electrode-tissue contact. **c** Design of a 3D printed mock drug (fluorescein)-loaded tissue adhesive patch. **d** Ex vivo demonstration of the fluorescein-loaded patch adhered to porcine skin. After removal of the adhered patch from the skin, fluorescence from the fluorescein released in the 3D printed spatial pattern can be observed. Cross-sectional micrographs show the diffusion of fluorescein from the patch into the skin over time. **e** Design of a tissue adhesive patch with fillable drug reservoirs. **f** Release of the mock drug (blue dye solution) into a gelatin hydrogel through 3D printed interfaces with differing pore size.

Beyond the fabrication of functional patches for defect repair, the 3D printing platform provides immense versatility for constructing multimaterial, tissue-interfacing devices (Fig. 6). Most biomedical devices typically require sutures, tacks, or a separate adhesive layer to affix onto tissues. Although various adhesives have been developed for interfacing devices, most existing adhesive hydrogels require separate synthesis processes that are incompatible with advanced device manufacturing techniques, such as 3D printing. In contrast, the 3D printable adhesive enables devices to directly incorporate a tissue adhesive interface during the additive manufacturing process. To explore the potential technologies enabled by the 3D printing platform, we designed several proof-of-concept devices for applications including bioelectronics and drug delivery. For instance, consider the case of a flexible, 3D printed bioelectronic patch featuring a simple LED circuit (Fig. 6a). The pattern of the tissue adhesive layer contains negative space designed for the conductive electrodes to interface with the tissue, ensuring stable adhesion around their perimeters and securing electrode-to-tissue contact. When the patch is adhered to an ex vivo porcine heart connected to a power supply, the LEDs remain illuminated through dynamic movement of the heart (Fig. 6b). This prototype demonstrates potential opportunities to print conformable bioelectronic devices for sensing or stimulating electrophysiological signals^41^. Next, we explore two illustrative concepts for achieving localized drug delivery using the 3D printing platform to fabricate tissue adhesive, drug-loaded systems. In one example, a mock drug (fluorescein) is directly loaded into the tissue adhesive ink, enabling precise spatial patterning of a drug-releasing patch (Fig. 6c). To visualize the release of fluorescein from the patch, a prototypical 3D printed patch was adhered to ex vivo porcine skin. At sequential timepoints, a small cross-sectional slice of the adhered system was cut and observed using fluorescence microscopy to capture the diffusion profile of the fluorescein into the skin (Fig. 6d). In this case, the intimate consolidation between the patch and the skin allows spatially focused delivery of the mock drug into the tissue at the site of adhesion. In another example, the tissue adhesive ink is used to print the interfacial layer of a multi-material drug reservoir (Fig. 6e). For this system, a therapeutic agent may be dispersed into a separate liquid or hydrogel matrix, then loaded into the adhesive reservoirs. As a visual demonstration, a mock drug solution containing blue dye was injected into the reservoirs using a syringe, and the diffusion of the dye into a gelatin hydrogel adherend was photographed to observe the release of the mock drug through the adhesive interface (Fig. 6f). These prototypes illustrate the potential utility of the 3D printable tissue adhesive for localized drug delivery, which is compelling for reducing the need for high-dosage systemic delivery of pharmacological substances. In this context, the 3D printable tissue adhesive could be favorable for mitigating device displacement and leakage to the surrounding tissues. Further design refinement and optimization regarding the biocompatibility, stability, and long-term functional efficacy of each of these concepts would be required to bring them beyond the proof-of-concept stage.

## Discussion

Taken together, the development of a 3D printable tissue adhesive signifies new opportunities in technologies for tissue repair and device fabrication. Ex vivo, in vitro, and in vivo studies demonstrate the promising wound sealing capabilities of the material, along with its favorable biocompatibility and broad potential to be used for applications such as blood-resistant adhesion, bioelectronics, and drug delivery. More generally, the unique material design offers a scalable strategy for preparing an off-the-shelf tissue adhesive with ease of processability for 3D printing or other fabrication methods. Still, more comprehensive biological studies would be required to fully assess the clinical efficacy of the 3D printed patches and devices and to elucidate the influence of tuning various properties (e.g., stress-strain behavior) on adhesion behavior and biological response. Looking ahead, the versatile platform proposed in this work has the potential to expand the design space for creating tunable, application-specific tissue adhesive structures.

## Methods

### Protocol approval

All animal studies were approved (protocol 1119-074-22) under the guidelines of the MIT Committee on Animal Care (CAC) and all surgical procedures and postoperative care were supervised by the MIT Division of Comparative Medicine veterinary staff.

### Materials

All chemicals were obtained from Sigma Aldrich unless otherwise stated and used without further purification. For synthesis of the 3D printable tissue adhesive, acrylic acid (AA), hydrophilic polyurethane (PU, HydroMed D3, AdvanSource Biomaterials), benzophenone, α-ketoglutaric acid, *N-*Hydroxysuccinimide (NHS), and 1-Ethyl-3-(3-dimethylaminopropyl) carbodiimide (EDC) were used. All porcine tissues used for ex vivo experiments were purchased from a research-grade tissue vendor (Sierra Medical Inc.).

### Grafting of poly(acrylic acid) to polyurethane

A precursor solution was prepared by combining 32 w/v % AA, 8 w/v % PU, 20 w/v % vacuum-degassed deionized water, and 40 w/v % ethanol and stirring until the PU was fully dissolved. 1.1 w/v % benzophenone and 0.1 w/v % α-ketoglutaric acid were added to the precursor solution and homogeneously mixed, then transferred to a sealed glass vial and cured in a UV crosslinker (364 nm, 15 W power) for 120 min. Benzophenone functions as a Type II free radical photoinitiator that enters an excited triplet state under UV irradiation, generating radical sites in PU (for example, by abstracting hydrogen from carbon-hydrogen containing molecules along the polyether backbone). These radical sites may then react with acrylic acid, initiating the growth of PU-grafted PAA chains (Supplementary Fig. 18). Benzophenone ketyl radicals can eventually combine with each other to form benzopinacol, which is removed during the dialysis process. For dialysis, the cured product was transferred to a cellulose membrane (Sigma Aldrich, typical molecular weight cut-off = 14,000) and purified in a pure ethanol bath for 24 h (replacing the ethanol every 12 h) followed by in a deionized water bath for 24 h (replacing the water every 12 h) with continuous magnetic stirring. The purified PU-PAA was cut into small pieces and dried in a desiccating oven at 70 °C for 48 h.

### Preparation of the 3D printable tissue adhesive ink

The dried PU-PAA was redissolved at a concentration of 20 w/w % in 70% ethanol and mixed in a 25:2 (v/v) ratio with a solution comprising 33.3 w/v % EDC and 33.3 w/v % NHS in 70% ethanol to yield around 10% (mol/mol) NHS functionalization of the carboxyl groups. The combined solution was then mixed in a 10:3 (v/v) ratio with 20 w/w % PU in 95% ethanol. To mitigate hydrolysis of NHS, the ink was prepared directly before use.

### 3D printing procedure

3D printing of the tissue adhesive ink and other polymer solutions was performed using a custom-designed 3D printer with a Cartesian gantry system (Aerotech). Under air pressure, inks were extruded from 5 mL syringe barrels through nozzles ranging in size from 50 to 200 µm (EFD Nordson). Printing paths were designed using Adobe Illustrator and CADFusion (Aerotech), then translated into G-code using a custom Python script. To achieve continuous printing of the tissue adhesive ink, we selected a printing pressure of 250 kPa (Ultimus V, Nordson EFD) and printing speeds ranging from 500−1800 mm/min. The structures were printed onto a glass slide (Corning) treated with hydrophobic coating (Rain-X). After printing, the structures were completely dried and sealed in plastic bags with desiccant (silica gel packets) before use.

### FTIR

32 scans were recorded for droplets of polymer on a Bruker Alpha II FT-IR spectrometer with a monolithic diamond crystal at a resolution of 4 cm^-1^. An equal number of background scans were recorded on air prior to each sample measurement. For analysis, each spectrum was normalized based on the peak around 2900 cm^-1^. As shown in Supplementary Fig. 4, the stretching bands of the C = O bond and the O-H bond in carboxylic acid can be clearly observed at around 1710 cm^-1^ and 3300 cm^-1^ for the samples containing PAA, indicating the retention of PAA via grafting and entanglement. The stretching and bending vibration bands of the C-NH bond on O = C-NH-C at around 3350 cm^-1^ and 1530 cm^-1^ and the stretching vibration band of the N-H bond at around 1700 cm^-1^ can also be observed for the PU-containing spectra, indicating the presence of a urethane group^42^. The strong stretching vibration band associated with the ether bond at 1100 cm^-1^ reflects the polyether character of the ether-based PU.

### ^1^H NMR

Proton (^1^H) nuclear magnetic resonance (NMR) spectra were measured at 400 MHz on a Bruker Avance III DPX 400. Approximately 100 mg of each sample were dissolved in 500 μL deuterated dimethylsulfoxide (DMSO-*d6*) for analysis. To evaluate the ratios of PAA:PU and NHS:PAA, each spectrum was normalized based on the peak around 2.22 ppm attributed to the methylene peak in PAA. The peaks at 7.06 ppm, attributed to the nitrogen-attached hydrogen in PU, were integrated with respect to the normalized peaks to approximate the molar ratios of PAA:PU in the final purified products (Supplementary Fig. 5). The NHS alkyl peaks at 2.76 ppm were integrated with respect to the normalized peaks to estimate the degree of NHS functionalization (Fig. 2d).

### Adhesion characterization

Adhesion tests were performed on in the inner surface of porcine skin washed with phosphate buffer solution (PBS). 3D printed tissue adhesive samples were adhered by applying gentle pressure upon the tissue substrate for 10 s. Commercial sealants were applied according to manufacturer instructions. Unless otherwise indicated, adhesion characterizations were performed 20-30 minutes after initial application to allow for swelling of the tissue adhesive material. Adhered samples were covered with gauze soaked in PBS to maintain a wet environment prior to measurement.

To measure interfacial toughness, tissue samples with widths of 2 cm were adhered to the various adhesives and tested via the standard T-peel test (ASTM F2256) using a mechanical testing machine (2.5 kN load cell, Zwick/Roell Z2.5). Data was collected using testXpert Testing Software. All tests were conducted with a constant peeling speed of 50 mm min^-1^. The measured force reached a plateau as the peeling process entered steady state. Interfacial toughness was determined by dividing two times the plateau force by the width of the tissue sample (Supplementary Fig. 8a). Hydrophilic nylon filters (1 µm pore size, TISCH Scientific) were used as a stiff backing for the 3D printed tissue adhesive.

To measure shear strength, tissue samples with an adhesion area of 2 cm x 2 cm were joined using the various adhesives and tested via the standard lap shear test (ASTM F2255) using a mechanical testing machine (2.5 kN load cell, Zwick/Roell Z2.5). All tests were conducted with a constant peeling speed of 50 mm min^-1^. Shear strength was determined by dividing the maximum force by the adhesion area (Supplementary Fig. 8b).

To measure burst pressure, 3 mm holes were introduced in 2.5 cm x 2.5 cm pieces of porcine skin using biopsy punches based on the ASTM F2392-04 standard defect size for burst pressure measurement. The holes were then sealed using 1.5 x 1.5 cm samples of the 3D printed tissue adhesive, or an equivalent area of Coseal, TachoSil, or Tegaderm. The size was determined based on previously reported measurements of burst pressure strength of surgical sealants^32^. The samples were fixed in a testing rig and PBS was injected at a constant rate of 5 mL min^-1^ to the point of failure (i.e., fluid leakage). Pressure was recorded by a pressure transducer (PX409, Omega) using OMEGA PC software. The burst pressure was determined as the maximum pressure upon which a leakage formed (modified ASTM F2382-04; Supplementary Fig. 8c).

### Rheological characterization

Rheological measurements of the tissue adhesive inks were performed using a rotational rheometer (AR-G2, TA instrument) with 40-mm diameter 2° steel cone geometry at 25 °C. Data was collected using TA Rheology Advantage. Apparent viscosity was measured as a function of shear rate using a continuous ramp over a logarithmic sweep from shear rate 1 to 1000 s^-1^. Shear storage modulus (G’) was measured as a function of shear stress using an oscillatory procedure with 1 Hz frequency over a logarithmic sweep from shear stress 1 to 10000 Pa. For all measurements, an aqueous solvent trap was used to minimize ink drying.

### Mechanical characterization

The tensile properties of mesh samples were measured following the standard tensile test (ASTM D412) using a mechanical testing machine (2.5 kN load cell, Zwick/Roell Z2.5). Samples were soaked for 20-30 min in PBS at 37 °C before measurement. Effective Young’s moduli were determined as the initial slope on the stress-strain curve.

### Microscope imaging

Microscopic 3D printed structures were imaged using an epifluorescence microscope (Nikon Eclipse LV100ND). Confocal microscope images of the mesh structure were obtained by an upright confocal microscope (SP 8, Leica) with 360 nm excitation wavelength for blue fluorescent beads. ImageJ (version 2.1.0) was used for image processing and analysis.

### Histology of porcine tissues

Porcine tissues samples (skin, tendon, aorta, and rectal sheath) were procured from Sierra Medical Inc. and fixed in 10% formalin for 24 h for histological analyses. Fixed tissue samples were placed into 70% ethanol and submitted for histological processing and H&E staining at the Hope Babette Tang (1983) Histology Facility in the Koch Institute for Integrative Cancer Research at the Massachusetts Institute of Technology. One sample for each tissue type was processed for histology.

### Fabrication of the collagen fiber-inspired patches

A silicone elastomer ink was prepared by mixing Dragon Skin 30 (Smooth-On) and SE 1700 (Dow Corning) together. Specifically, Dragon Skin 30 part A, Dragon Skin 30 part B, SE 1700 base, and SE 1700 catalyst were added in a 10:10:10:1 weight ratio and mixed thoroughly using a Thinky mixer (AR-100, Thinky). The ink was printed onto a glass slide (Corning) treated with a hydrophobic coating (RainX) into the desired geometry and cured in the oven at 120 °C for 30 min. After curing and cooling, a layer of the tissue adhesive ink (prepared as described above) was printed on top of the silicone layer, following the same geometry. After drying, the patch was removed from the glass slide and evaluated using a standard tensile test.

### Fabrication of the liquid-infused blood resistant patch

Tissue adhesive ink was prepared as described above and printed onto a glass slide (Corning) treated with hydrophobic coating (RainX) into a 25 cm by 25 cm repeating lattice pattern with filament width ~150 µm and gap width ~150 µm. After drying, the adhesive structure was integrated with a collagen wound dressing sponge (Puracol) of the same dimensions by exposing the uppermost surface of the printed structure to steam, allowing the surface to become slightly hydrated, then immediately placing the collagen sponge on the hydrated surface to allow crosslinking between the two substrates. The integrated patch was removed from the glass slide and infiltrated with oleic acid. To demonstrate blood-resistant tissue adhesion, samples of the oil-infused patch were applied to porcine skin samples covered with heparinized porcine blood (Lampire Biological Laboratories, Inc.) using gentle pressure for 10–30 s.

### In vitro biocompatibility

To evaluate the in vitro biocompatibility and cytotoxicity of the mesh, a LIVE/DEAD assay was used to assess BALB/c 3T3 clone A31 mouse fibroblasts (American Type Culture Collection®; CCL163™). To prepare conditioned media, 500 mg of Coseal, TachoSil, and the 3D printed tissue adhesive were each incubated in 10 ml of Dulbecco’s modified Eagle’s medium (DMEM) supplemented with 10 v/v % fetal bovine serum and penicillin-streptomycin (100 U mL^−1^) at 37 °C for 24 h. The supplemented DMEM (without any material incubation) was used as a control. 3T3 cells were plated in confocal dishes (20-mm diameter) at a density of 0.5 × 10^5^ cells cm^−2^ (*N* = 4 for each group). The cells were then treated with either conditioned or control media and incubated at 37 °C for 24 h in a 5% CO_2_ atmosphere. Cell viability was determined by a LIVE/DEAD viability/cytotoxicity kit for mammalian cells (Thermo Fisher Scientific). A confocal microscope (SP 8, Leica) was used to image live cells with excitation/emission at 495 nm/515 nm and dead cells at 495 nm/635 nm, respectively. Cell viability was calculated by counting live (green fluorescence) and dead (red fluorescence) cells using ImageJ (version 2.1.0).

### In vivo biocompatibility

Female Sprague-Dawley rats (225-250 g, Charles River Laboratories) were used for all in vivo studies. Before implantation, the 3D printed patch was exposed for 1 h under UV light. Commercially available tissue adhesives were used as provided in sterile packages following the provided user guide or manual for each product.

For implantation in the dorsal subcutaneous space, rats were anesthetized using isoflurane (2-3% isoflurane in oxygen) in an anesthetizing chamber. Anesthesia was maintained using a nose cone. The back hair was removed, and the animals were placed over a heating pad for the duration of the surgery. The subcutaneous space was accessed by a 1–2 cm skin incision per implant in the center of the animal’s back. To create space for implant placement, blunt dissection was performed from the incision towards the animal shoulder blades. Samples of the 3D printed patch and TachoSil with the size of 20 mm in width and 20 mm in length were placed in the subcutaneous pocket created above the incision without detachment. The incision was closed using interrupted sutures (4-0 Vicryl, Ethicon) and 3–6 mL of saline was injected subcutaneously. Up to four implants were placed per animal ensuring no overlap between each subcutaneous pocket created. 2 or 4 weeks after the implantation, the animals were euthanized by CO_2_ inhalation. Subcutaneous regions of interest were excised and fixed in 10% formalin for 24 h for histological analyses. Fixed tissue samples were placed into 70% ethanol and submitted for histological processing and H&E or Masson’s trichrome (MT) staining at the Hope Babette Tang (1983) Histology Facility in the Koch Institute for Integrative Cancer Research at the Massachusetts Institute of Technology. Representative histology images of each group were shown in the corresponding figures. The subcutaneous implantation model was performed on 3 independent samples with similar results.

### In vivo rat trachea defect repair

For the in vivo trachea defect repair model, rats were anesthetized using isoflurane (2-3% isoflurane in oxygen) in an anesthetizing chamber. Anesthesia was maintained using a nose cone. Hair covering the throat area was removed, and the animals were placed over a heating pad for the duration of the surgery. The trachea was exposed by a 2 cm midline skin incision followed by separation of the sternohyoid and sternothyroid muscles. A longitudinal oval-shaped defect was created by using a 1 mm-diameter biopsy punch to create two adjacent holes in the trachea. A 3D printed patch or TachoSil patch with the size of 5 mm in width and 10 mm in length was applied over the defect by gently pressing with a sterile cotton tip applicator for 10–30 s. After adhesion, leakage from the sealed defect was tested by introducing warm saline solution and checking for bubbles. Following confirmation of an air-tight seal, the muscle and skin layers were closed with sutures (4-0 Vicryl, Ethicon). 2, 4, or 6 weeks after the surgery, the animals were euthanized by CO_2_ inhalation. Tracheal regions of interest were excised and fixed in 10% formalin for 24 h for histological analyses. Fixed tissue samples were placed into 70% ethanol and submitted for histological processing and H&E or MT staining at the Hope Babette Tang (1983) Histology Facility in the Koch Institute for Integrative Cancer Research at the Massachusetts Institute of Technology. Representative histology images were shown in the corresponding Figs. 1, 3, and 6 weeks after surgery, the animals were imaged using Micro-CT. The trachea defect repair model was performed on 3 independent samples with similar results.

### Micro-CT imaging of rat tracheas

Micro-CT was performed at the Preclinical Modeling, Imaging, & Testing Core (PMIT) at the Koch Institute. Micro-CT images were obtained using a SkyScan 1276 (Bruker). To perform Micro-CT imaging, rats were anesthetized using isoflurane (1.5-2.5%) and transferred to an animal holder that was mounted into the scanner. Scans were obtained using the following parameters: pixel size 40.2 µm, source voltage 85 kV, source current 200 µA, exposure 179 ms, rotation step 230.5 mm, no frame averaging, and aluminum filter (1 mm). The images were reconstructed using NRecon software (Bruker). Luminal perimeter fraction was computed using ImageJ to measure the ratio of the injured trachea cross-sectional perimeter to the non-injured perimeter for each animal. Luminal area fraction was computed using ImageJ (version 2.1.0) to measure the ratio of the injured trachea cross-sectional area to the non-injured area for each animal. After the conclusion of the 6 week timepoint, the animals were euthanized by CO_2_ inhalation.

### In vivo rat colon defect repair

For the in vivo colon defect repair model, rats were anesthetized using isoflurane (2-3% isoflurane in oxygen) in an anesthetizing chamber. Anesthesia was maintained using a nose cone. Abdominal hair was removed, and the animals were placed over a heating pad for the duration of the surgery. The colon was exposed by a median laparotomy. The exposed colon was packed with moistened sterile gauzes before creating a defect to prevent contamination of the abdominal cavity. A 10 mm incision was made to the colon using scissors and repaired using a 3D printed patch (10 mm in width and 20 mm in length) or sutures. After repair, warm saline was injected into the colon by a 32-gauge needle syringe to confirm formation of a fluid-tight seal. The muscle and skin layers were closed with sutures (4-0 Vicryl, Ethicon). 2 or 4 weeks after the surgery, the animals were euthanized by CO_2_ inhalation. Regions of interest were excised and fixed in 10% formalin for 24 h for histological analyses. Fixed tissue samples were placed into 70% ethanol and submitted for histological processing and H&E or MT staining at the Hope Babette Tang (1983) Histology Facility in the Koch Institute for Integrative Cancer Research at the Massachusetts Institute of Technology. Representative histology images were shown in the corresponding figures. The colon defect repair model was performed on 3 independent samples with similar results.

### In vivo rat liver defect repair

For the in vivo rat liver defect repair model, rats were anesthetized using isoflurane (2-3% isoflurane in oxygen) in an anesthetizing chamber. Anesthesia was maintained using a nose cone. Abdominal hair was removed, and the animals were placed over a heating pad for the duration of the surgery. The liver was exposed by a laparotomy. An injury 5 mm in length and 2 mm in depth was made to the liver using a surgical scalpel. To seal the injury, a liquid-infused patch measuring around 2 cm by 3.5 cm was placed onto the bleeding defect site and gently pressed for 10 s. To confirm hemostasis, the region was washed with saline and observed for 30 min to check for any signs of further blood loss. At the termination of the study, the animals were euthanized by CO_2_ inhalation. The liver defect repair model was performed on 2 independent samples with similar results.

### In vivo rat femoral artery defect repair

For the in vivo rat femoral artery defect repair model, rats were anesthetized using isoflurane (2-3% isoflurane in oxygen) in an anesthetizing chamber. Anesthesia was maintained using a nose cone. Leg hair was removed, and the animals were placed over a heating pad for the duration of the surgery. The femoral artery was exposed via an incision into the thigh. A snip around 2 mm in length was made to the artery using surgical scissors. To seal the injury, a liquid-infused patch measuring around 2 cm by 2 cm was placed onto the bleeding defect site and gently pressed for 10 s. To confirm hemostasis, the region was washed with saline and observed for 30 min to check for any signs of further blood loss. At the termination of the study, the animals were euthanized by CO_2_ inhalation. The femoral artery defect repair model was performed on 2 independent samples with similar results.

### Fabrication of the adhesive bioelectronic patch

Tissue adhesive ink was prepared as described above and printed in a pattern featuring circular void spaces for electrodes onto a glass slide treated with hydrophobic coating. For the insulator layer, 20 w/v % polyurethane (HydroThane AL93A, AdvanSource biomaterials) dissolved in 1:1 tetrahydrofuran (THF) and dimethylformamide (DMF) was prepared and printed over the adhesive layer (400 µm-diameter printing nozzle, 200 kPa, 500 mm min^-1^). Next, silver conductive ink was used to print the electrodes and circuitry (100 µm-diameter printing nozzle, 50 kPa, 800 mm min^-1^). LEDs were attached to the circuit using a small amount of the silver ink. After drying, the bioelectronic patch was removed from the glass slide and adhered to an ex vivo porcine heart and a power source was used to run a current through the tissue to confirm illumination of the LEDs.

### Fabrication of the mock drug delivery patches

To prepare the fluorescent mock drug-loaded ink, tissue adhesive ink was prepared as described above and mixed with a small amount of fluorescein. The non-fluorescent and fluorescent inks were printed in corresponding positive and negative patterns onto a glass slide treated with hydrophobic coating. After drying, the patch was removed from the glass slide and adhered to ex vivo porcine skin. At sequential timepoints, a small cross-sectional slice of the patch/tissue interface was cut and imaged using an epifluorescence microscope (Nikon Eclipse LV100ND) to capture the diffusion profile into the skin.

To prepare the drug reservoir patch, tissue adhesive ink was prepared as described above and printed with two different lattice dimensions at the reservoir interfaces. The reservoir walls were printed using three layers of an elastomer ink comprising 20 w/w % polyurethane (HydroThane AL93A, AdvanSource biomaterials) dissolved in 1:1 tetrahydrofuran (THF) and dimethylformamide (DMF). Before the walls were fully dried, spin-coated films of the HydroThane solution were cut to shape and attached to provide enclosures for the reservoirs. After drying, the patch was removed from the glass slide and adhered to a gelatin hydrogel. The mock drug solution (a mix of blue food dye and water) was injected into the reservoirs using a syringe needle, and the diffusion of the dye into the gelatin hydrogel was photographed over 5 h.

### Statistics & reproducibility

MATLAB (version R2021b) and Microsoft Excel (version 2310) were used to analyze all data in this work. Data distributions were assumed to be normal for all parametric tests but not formally tested. No statistical method was used to predetermine sample size. For the statistical analyses between two groups, statistical significance and *p* values were determined using a two-tailed Student’s t-test with unequal variance. The following significance thresholds were used: *ns p* > 0.05; * *p* ≤ 0.05; ** *p* ≤ 0.01; *** *p* ≤ 0.001.

### Reporting summary

Further information on research design is available in the Nature Portfolio Reporting Summary linked to this article.

### Supplementary information

Supplementary Information

Peer Review File

Description of Additional Supplementary Files

Supplementary Movie S1

Supplementary Movie S2

Reporting Summary

### Source data

Source Data

## Data Availability

The data supporting the findings of this study are available within the article and its supplementary files. Any additional requests for information can be directed to, and will be fulfilled by, the corresponding authors. Source data are provided with this paper.

## Code availability

The custom codes used in this study for 3D printing are available from the corresponding authors upon request.
