# Supplementary material for: A 3D printable tissue adhesive
Source: Nat Commun. 2024 Feb 9;15:1215. doi: 10.1038/s41467-024-45147-9 (PMC10853267; doi:10.1038/s41467-024-45147-9)
Supplement: Supplementary file 1 — Supplementary Information [file 41467_2024_45147_MOESM1_ESM.pdf]

Supplementary information for

A 3D printable tissue adhesive

Sarah J. Wu[1], Jingjing Wu[1], Samuel J. Kaser[2], Heejung Roh[1], Ruth D. Shiferaw[1], Hyunwoo Yuk[1,4]*, Xuanhe Zhao[1,3]*

[1] Department of Mechanical Engineering, Massachusetts Institute of Technology; Cambridge, Massachusetts, 02139, USA.
[2] Department of Chemistry, Massachusetts Institute of Technology; Cambridge, Massachusetts, 02139, USA.
[3] Department of Civil and Environmental Engineering, Massachusetts Institute of Technology; Cambridge, Massachusetts, 02139, USA.
[4] Present address: SanaHeal, Inc., Cambridge, MA, USA

*Corresponding authors. Emails: hyunwooyuk@sanaheal.com, zhaox@mit.edu

Contents

Supplementary Fig. 1 | Representative stress-strain curves of samples prepared using varying ratios of PU-PAA to PU in the 3D printing ink

Supplementary Fig. 2 | Images of the synthesized PU-PAA material and solutions of varying polymer content

Supplementary Fig. 3 | Stability of the PU-PAA polymer

Supplementary Fig. 4 | FTIR spectra for materials prepared using different precursor compositions

Supplementary Fig. 5 | ^{1}H NMR spectra for PU-PAA prepared using varying precursor ratios of acrylic acid (AA) and PU

Supplementary Fig. 6 | Swelling of the printed tissue adhesive material

Supplementary Fig. 7 | Swelling of an adhered 3D printed mesh

Supplementary Fig. 8 | Experimental setups characterizing adhesion performance

Supplementary Fig. 9 | Representative burst pressure curves of 3D printed patches with and without backing layer

Supplementary Fig. 10 | Representative stress-strain curves for 3D printed samples with varying gap width (w) to filament diameter (d) ratios

Supplementary Fig. 11 | Representative stress-strain curves for 3D printed samples with different alignment angles between filaments

Supplementary Fig. 12 | In vitro biocompatibility

Supplementary Fig. 13 | In vitro degradation of PU-PAA

Supplementary Fig. 14 | In vivo wound sealing of tracheal defects in rats

Supplementary Fig. 15 | Micro-CT observation of tracheal healing in rats

Supplementary Fig. 16 | In vivo wound sealing of colonic defects in rats

Supplementary Fig. 17 | Wetting configurations and energy criteria for the liquid-infused adhesive system comprising air, the tissue adhesive material, oleic acid, and tissue

Supplementary Fig. 18 | Proposed reaction mechanism of benzophenone

Supplementary Discussion 1 | Stability of the liquid-infused patch

Supplementary References

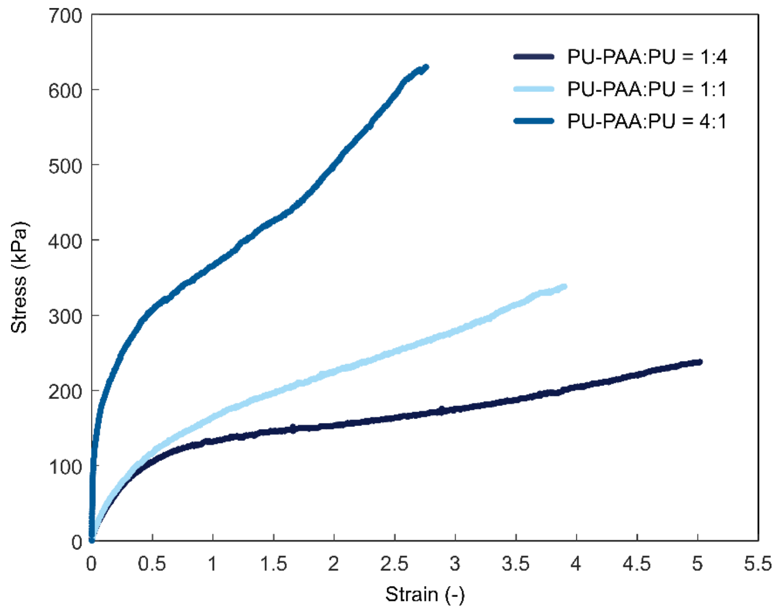

Supplementary Fig. 1 | Representative stress-strain curves of samples prepared using varying ratios of PU-PAA to PU in the 3D printing ink. Samples containing different weight ratios of PU-PAA to PU were 3D printed and their tensile properties were measured in their as-printed dry state.

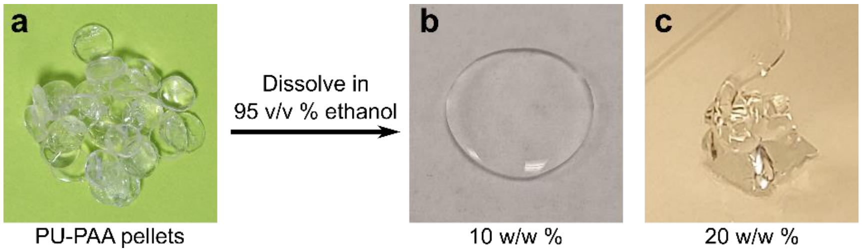

Supplementary Fig. 2 | Images of the synthesized PU-PAA material and solutions of varying polymer content. a, PU-PAA pellets obtained after synthesis, purification, and drying. **b-c**, PU-PAA solutions of 10 w/w % (b) and 20 w/w % (c) concentrations. As the polymer concentration increases, the inks transition from spreading freely to having suitable yield stress for 3D printing.

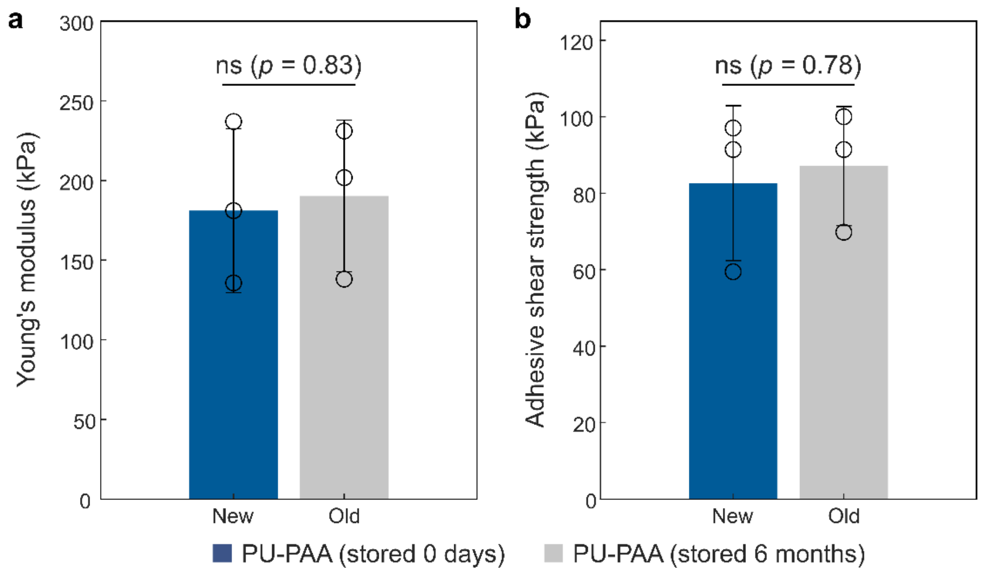

Supplementary Fig. 3 | Stability of the PU-PAA polymer. a, Young's modulus and **b,** adhesive shear strength of samples printed using newly synthesized PU-PAA and PU-PAA stored in ambient conditions for 6+ months. Values represent the mean and standard deviation (n = 3 independent samples). Statistical significance and p values were determined using a two-tailed Student's t-test with unequal variance: *ns* indicates $p > 0.05$.

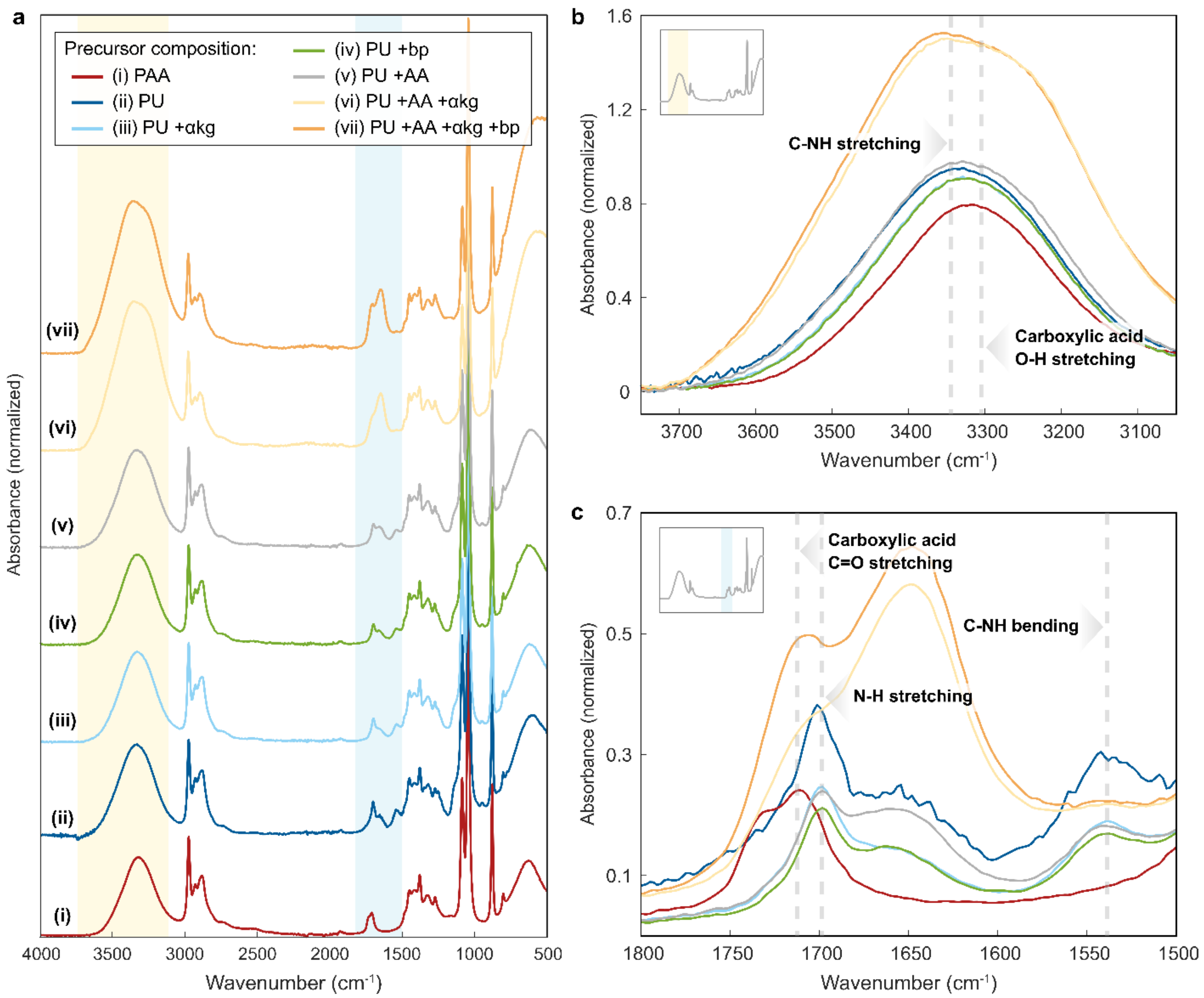

Supplementary Fig. 4 | FTIR spectra for materials prepared using different precursor compositions. a, Full FTIR spectra for samples prepared from precursors containing (i) PAA alone, (ii) PU alone, (iii) PU and *α*-ketoglutaric acid (*α*-kg), (iv) PU and benzophenone (bp), (v) PU with acrylic acid (AA) and no initiator, (vi) PU with AA and *α*-kg, and (vii) PU with AA, *α*-kg, and bp. Each spectrum is normalized based on the peak at 2900 cm^{-1}. **b,** Zoomed-in spectra for a frequency range of 3700-3100 cm^{-1}. **c,** Zoomed-in spectra for a frequency range of 1800-1500 cm^{-1}.

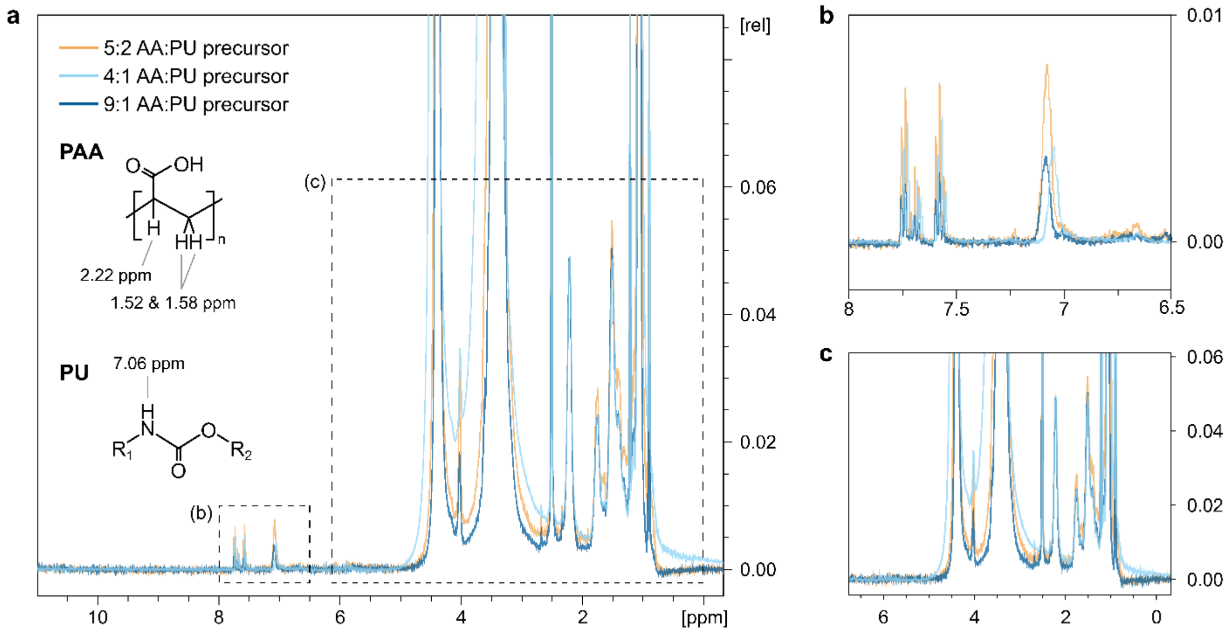

Supplementary Fig. 5 | ^{1}H NMR spectra for PU-PAA prepared using varying precursor ratios of acrylic acid (AA) and PU. Precursor solutions containing varying AA:PU weight ratios of 5:2, 4:1, and 9:1 PAA:PU were used to synthesize PU-PAA. The final products were purified to remove unincorporated monomers and oligomers, then characterized using ^{1}H NMR. **a,** Full NMR spectra of all three samples normalized based on the peak around 2.22 ppm (attributed to PAA). **b,** Zoomed-in spectra from 8 to 6.5 ppm. The peak at 7.06 ppm is attributed to the nitrogen-attached hydrogen in PU. **c,** Zoomed-in spectra from 6 to 0 ppm.

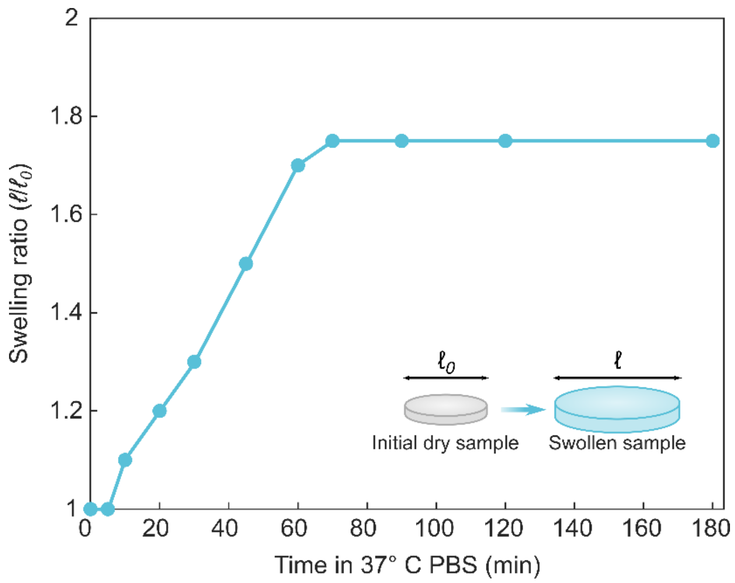

Supplementary Fig. 6 | Swelling of the printed tissue adhesive material. Swelling ratio of a disk-shaped tissue adhesive sample vs. time immersed in PBS at 37° C (initial diameter l_0 = 10 mm). Three independent experiments were conducted with similar results.

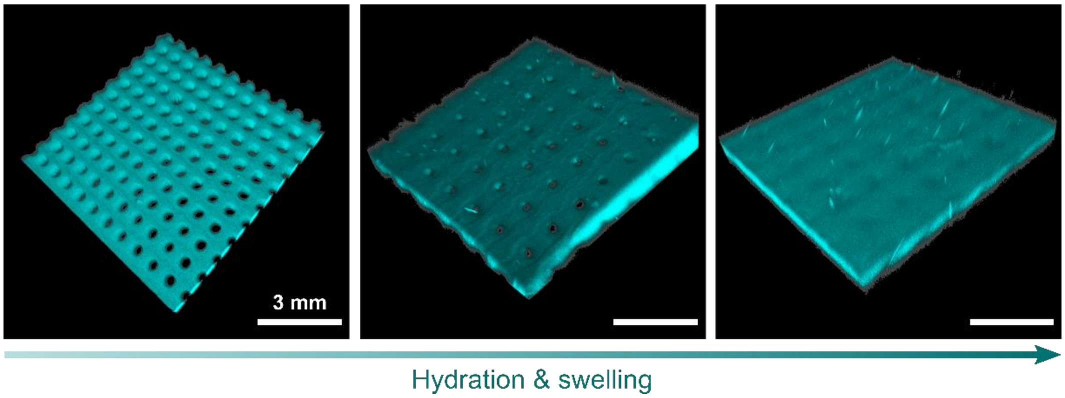

Supplementary Fig. 7 | Swelling of an adhered 3D printed mesh. To visualize swelling using fluorescent confocal microscopy, the tissue adhesive ink was mixed with blue fluorescent latex microbeads prior to 3D printing. The lattice-patterned patch was adhered to a gelatin hydrogel and immersed in saline solution. Hydration and swelling of the mesh patch over the course of ~15 minutes led to visible shrinkage of the pores.

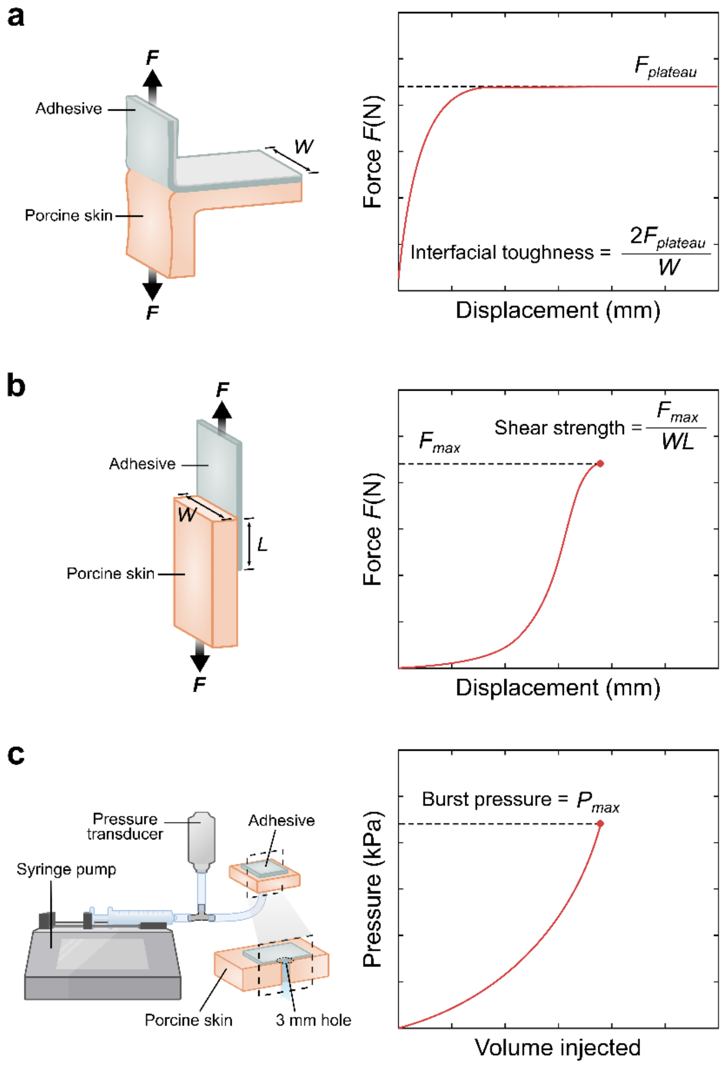

Supplementary Fig. 8 | Experimental setups characterizing adhesion performance. a, Schematic illustration of the experimental setup to measure interfacial toughness based on a 180-degree peel test (ASTM F2256). **b**, Schematic illustration of the experimental setup to measure adhesive shear strength based on a lap-shear test (ASTM F2255). **c**, Schematic illustration of the experimental setup to measure burst pressure based on ASTM F2392-04. Plots in a-c are generic drawings and do not represent real data.

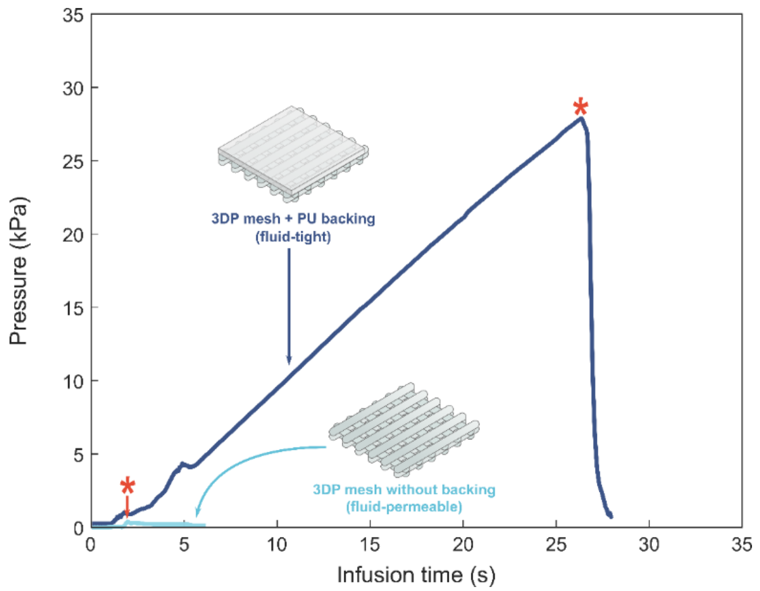

Supplementary Fig. 9 | Representative burst pressure curves of 3D printed patches with and without backing layer. Lattice patterns printed onto a thin layer of PU achieved fluid-tight seals with porcine skin, sustaining burst pressures exceeding 20 kPa (main Fig. 3c). Without backing the backing layer, the patches formed fluid-permeable adhesive interfaces. For the representative curves, * indicates the burst pressure.

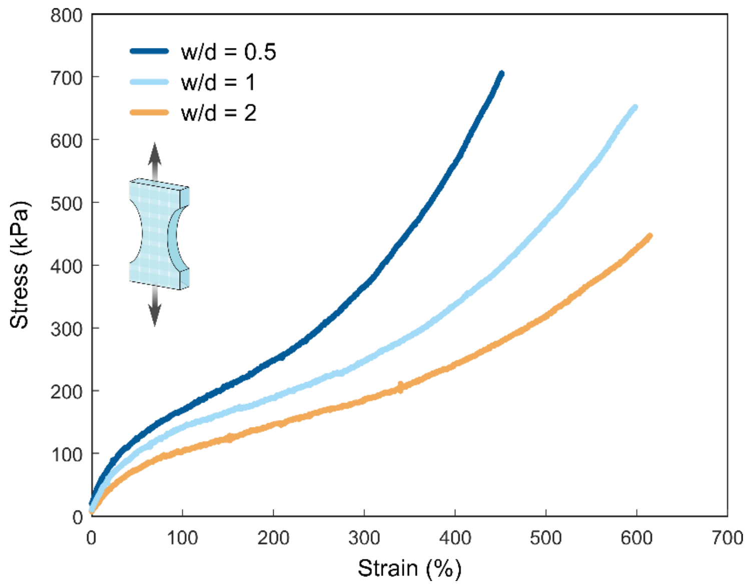

Supplementary Fig. 10 | Representative stress-strain curves for 3D printed samples with varying gap width (w) to filament diameter (d) ratios. Tensile tests of 3D printed tissue adhesive meshes with w/d ratios of 0.5, 1, and 2 were measured (with *d* fixed at 200 µm). All measurements were taken with the materials in a fully hydrated state, following submersion in PBS at 37 °C for 20-30 minutes.

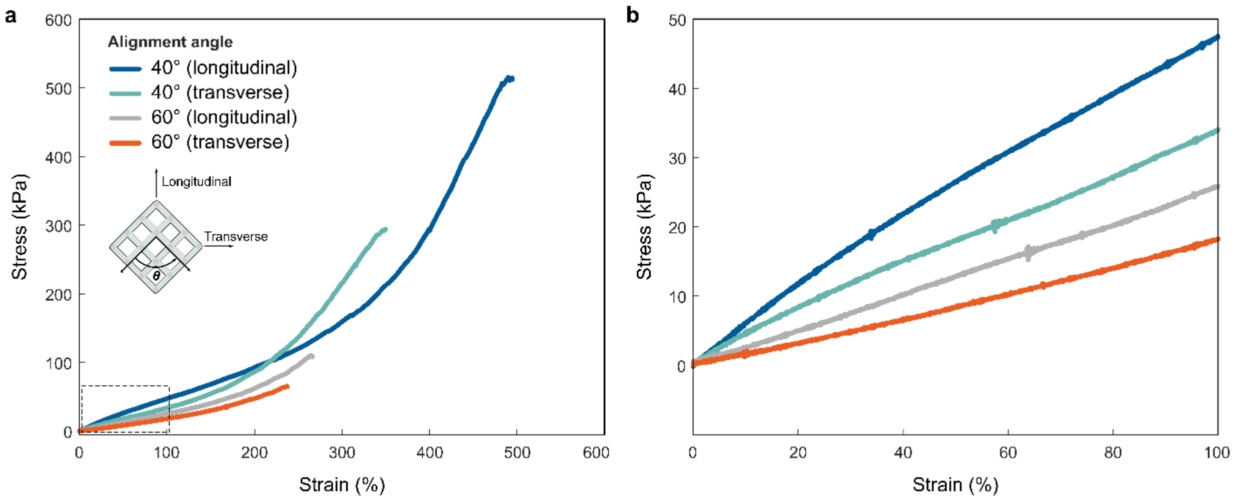

Supplementary Fig. 11 | Representative stress-strain curves for 3D printed samples with different alignment angles between filaments. a, Full stress-strain curves for samples with angles of alignment of 40° and 60°. **b,** Zoomed-in plot depicting the initial stress-strain slope of each representative sample. All measurements were taken with the materials in a fully hydrated state, following submersion in PBS at 37 °C for 20-30 minutes.

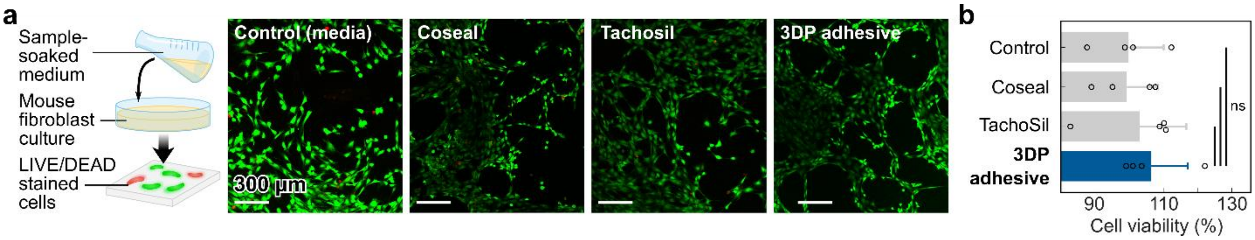

Supplementary Fig. 12 | In vitro biocompatibility. a, Representative in vitro LIVE/DEAD staining and **b,** quantitative cell viability analysis of 3T3 mouse fibroblasts cultured with Dulbecco's modified Eagle's medium (DMEM, control) and DMEM soaked with Coseal, TachoSil, and the 3D printed patch. Values and error bars represent the mean and standard deviation (n = 4 independent samples). Statistical significance and p values were determined using a two-tailed Student's t-test with unequal variance: *ns* indicates $p > 0.05$. Specific p values are provided in the Source Data file.

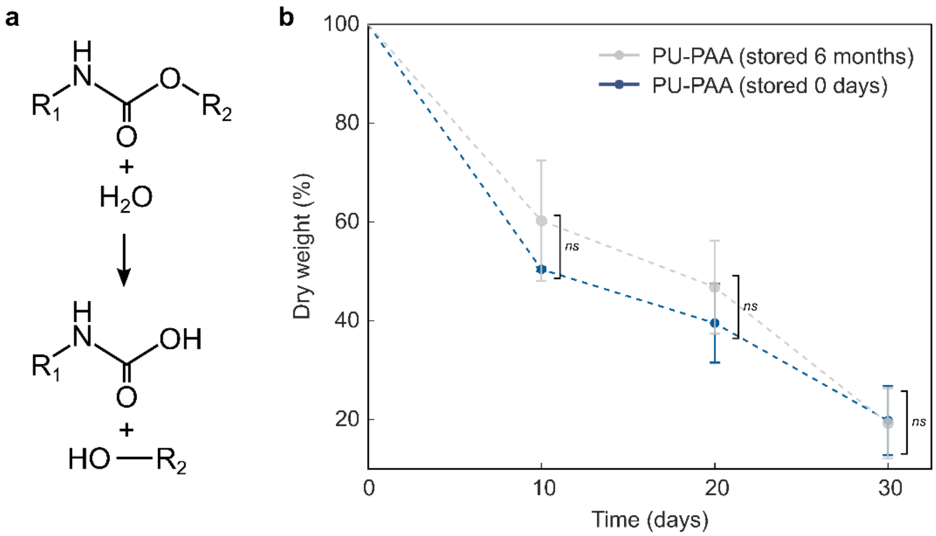

Supplementary Fig. 13 | In vitro degradation of PU-PAA. a, General hydrolytic degradation of polyurethane. **b,** Degradation profiles. Samples of PU-PAA were submerged in 37 °C PBS and shaken continuously at 60 rpm. At days 10, 20, and 30, the samples were removed, dried thoroughly at 70 °C, and weighed. No significant difference in degradation was found between freshly synthesized samples and samples which were stored in ambient conditions for 6 months prior. Values represent the mean and standard deviation (n = 3 independent samples). Statistical significance and p values were determined using a two-tailed Student's t-test with unequal variance: *ns* indicates $p > 0.05$. Specific p values are provided in the Source Data file.

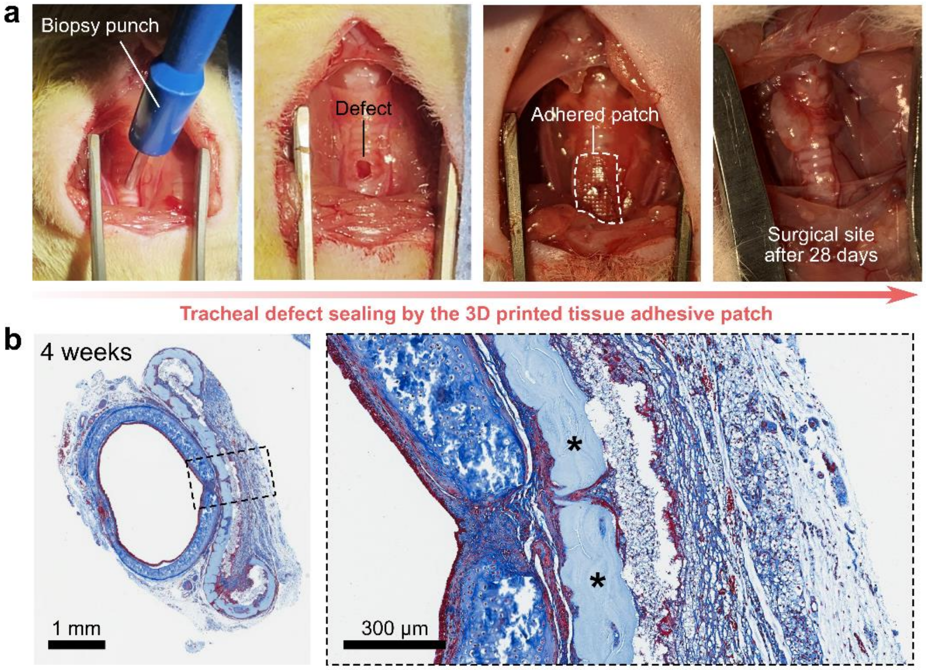

Supplementary Fig. 14 | In vivo wound sealing of tracheal defects in rats. a, Experimental photographs for the rat tracheal defect-repair model. A 1-mm diameter biopsy punch was used twice to create a 2 mm x 1 mm oval-shaped hole, which was sealed with a 3D printed patch. The defects remained sealed for 28 days post-surgery. **b,** Representative histology images of the tracheas harvested after 28 days stained with Masson's trichrome stain. * indicates the adhesive. Three independent experiments were conducted with similar results.

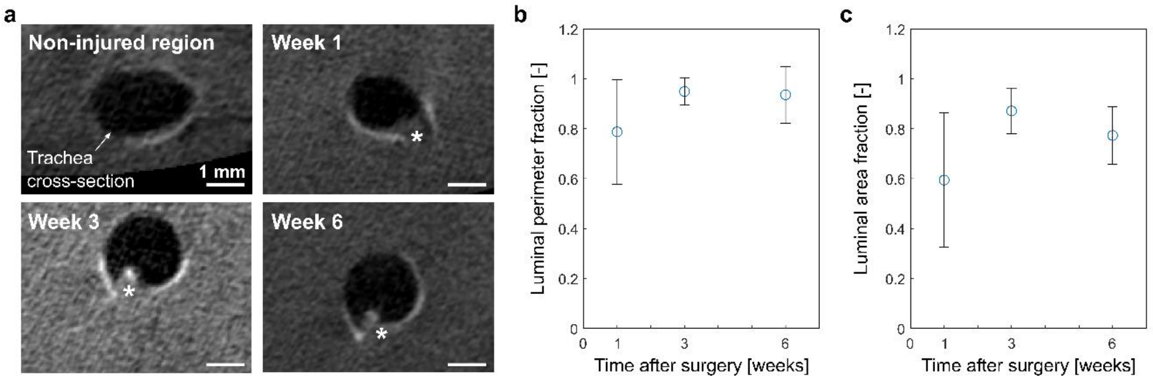

Supplementary Fig. 15 | Micro-CT observation of tracheal healing in rats. a, Representative micro-CT scans of an injured rat trachea repaired with a 3D printed patch. * indicates the defect site. **b,** Luminal perimeter fraction as a function of time after surgery. Luminal perimeter fraction was computed using ImageJ by measuring the ratio of the injured trachea cross-sectional perimeter to the non-injured perimeter for each animal. **c,** Luminal area fraction as a function of time after surgery. Luminal area fraction was computed using ImageJ by measuring the ratio of the injured trachea cross-sectional area to the non-injured area for each animal. Values represent the mean and standard deviation (n = 3 independent samples).

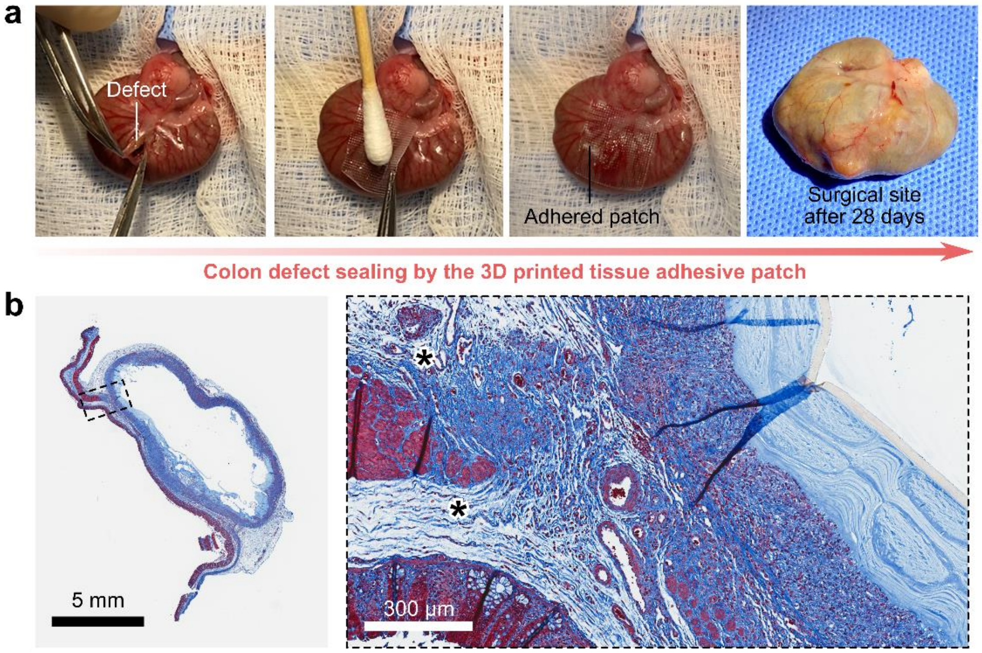

Supplementary Fig. 16 | In vivo wound sealing of colonic defects in rats. a, Experimental photographs for the rat colon defect-repair model. Surgical scissors were used to create a 10-mm incision, which was sealed with a 3D printed patch. The defects remained sealed for 28 days post-surgery. **b**, Representative histology images of the colons harvested after 28 days stained with Masson's trichrome stain. * indicates the adhesive. Three independent experiments were conducted with similar results.

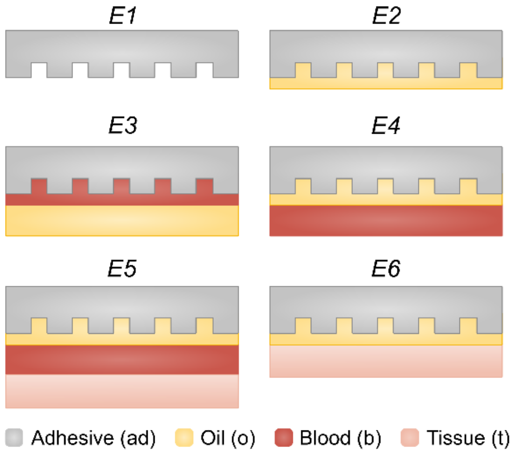

Desired energy relation:	Criteria:
$E1 > E2$	$\boldsymbol{r\gamma_{o/a}cos\theta_{o/ad(a)}} > \mathbf{0}$
$E3 > E4$	$r\left(\gamma_{o/a}cos\theta_{o/ad(a)} - \gamma_{b/a}cos\ \theta_{b/ad(a)}\right) > 0$
$E5 > E6$	$\gamma_{o/b} + \gamma_{o/a}cos\ \theta_{o/t(a)} - \gamma_{b/a}cos\ \theta_{b/t(a)} > 0$

Supplementary Fig. 17 | Wetting configurations and energy criteria for the liquid-infused adhesive system comprising air, the tissue adhesive material, oleic acid, and tissue. For the liquid-infused patch to be thermodynamically stable in air, the desired energy relation is E1 > E2. For the patch to be preferentially wetted by the oil in the presence of blood, the desired energy relation is E3 > E4. To ensure repelling of blood at the tissue surface, the desired energy relation is E5 > E6. Here, r is the ratio of the total surface area to the projected area, $\gamma_{x/y}$ is the interfacial energy between substances x and y (where o = oil, a = air, ad = adhesive, b = blood, and t = tissue), and $\theta_{x/y(z)}$ denotes the apparent contact angle of substance x on substance y in the presence of substance z. See Supplementary Discussion 1 for more details.

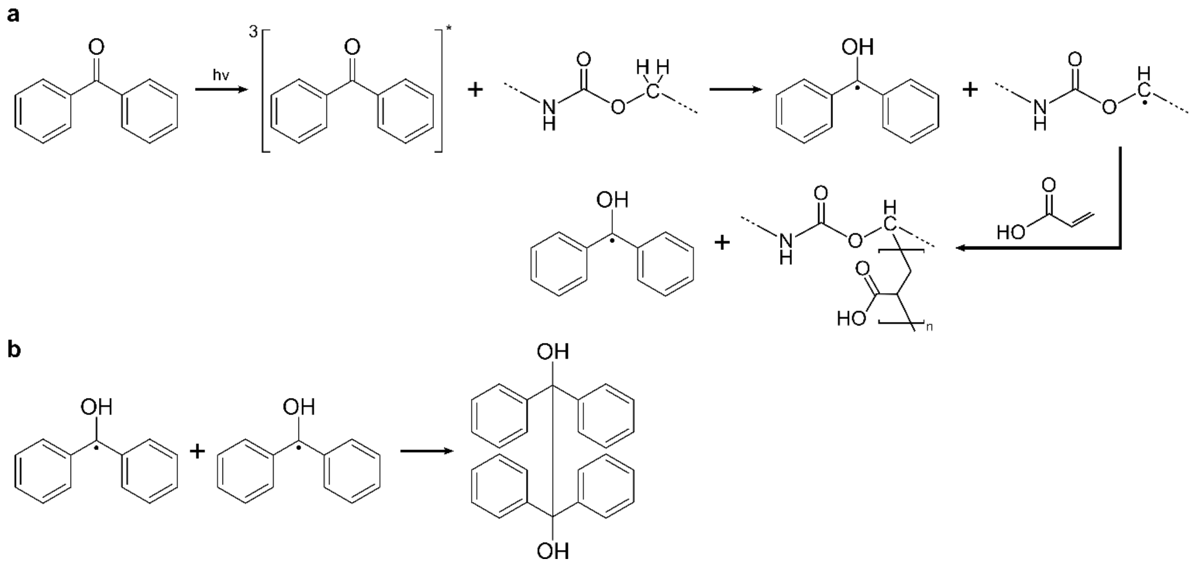

Supplementary Fig. 18 | Proposed reaction mechanism of benzophenone. a, Benzophenone is activated by UV light to enter a reactive triplet state, which can generate radical sites in the PU backbone (for example, by abstracting hydrogen from carbon-hydrogen containing molecules). These radical sites may then react with acrylic acid, initiating the growth of PU-grafted PAA chains. **b,** Combination of benzophenone ketyl radicals to form benzopinacol.

Supplementary Discussion: Stability of the liquid-infused patch

The stability of the liquid-infused patch is governed by the energy relations that describe the liquid-substrate contact line[2,3]. In the case of the textured 3D printed patch, the infusing liquid is held in place through a combination of van der Waals and capillary forces. To evaluate the thermodynamics of the system, we consider interactions between the four phases present: the oil (i.e., oleic acid), air, blood, and the tissue adhesive (**Supplementary Fig. 17**). Considering a square lattice-printed pattern of the tissue adhesive surface with filament width *a*, gap width *b*, and thickness *h*, the ratio of the total surface area to the projected area is given by:

$$r = 1 + \frac{4bh}{(b+a)^2} \tag{S1}$$

First, to determine the stable configuration of the liquid-infused 3D printed patch in air, we compare the total surface energies (denoted E_n) of Configurations E1 and E2. Configuration E1 describes a dry (i.e., not infiltrated) surface and Configuration E2 describes a fully encapsulated surface. For the oil to infiltrate the tissue adhesive, Configuration E2 should have a lower energy state than Configuration 1, such that:

$$E_1 > E_2 \tag{S2a}$$

This condition is equivalent to:

$$r\gamma_{ad/a} > r\gamma_{ad/o} \tag{S2b}$$

where $\gamma_{x/y}$ denotes the interfacial energy between substances *x* and *y*. *ad* denotes adhesive, *a* denotes air, and *o* denotes oil. Using Young's equation, this reduces to:

$$r\gamma_{o/a} cos\theta_{o/ad(a)} > 0 \tag{S2b}$$

where $\theta_{o/ad(a)}$ denotes the apparent contact angle of the oil on the adhesive substrate in air. Similarly, for the tissue adhesive patch to be preferentially wetted by the oil in the presence of blood, we consider Configurations E3 and E4:

$$E_3 > E_4 \Leftrightarrow r\left(\gamma_{o/a} cos\theta_{o/ad(a)} - \gamma_{b/a} cos\,\theta_{b/ad(a)}\right) > 0 \tag{S3}$$

Finally, we compare the surface energies of Configurations E5 and E6 to ensure repulsion of blood at the tissue interface. The corresponding energy relation that must be satisfied is:

$$E_5 > E_6 \Leftrightarrow \gamma_{o/b} + \gamma_{o/a} cos\,\theta_{o/t(a)} - \gamma_{b/a} cos\,\theta_{b/t(a)} > 0 \tag{S4}$$

The system energies can thus be approximated by plugging in the relevant contact angles, interfacial energies, and geometrical parameters ($r \approx 1.22$, $\gamma_{o/a} \approx 31.92$ mN m^{-1}, $\gamma_{b/a} \approx 72.0$ mN m^{-1}, $\gamma_{o/b} \approx 40$ mN m^{-1}, $\theta_{o/ad(a)} \approx 7.6°$, $\theta_{b/ad(a)} \approx 72.1°$, $\theta_{o/t(a)} \approx 4.2°$, $\theta_{b/t(a)} \approx 84°$)[4-7]. In doing so, it is evident that the thermodynamic conditions summarized in Supplementary Fig. 17 are satisfied.

Supplementary References

1. Lim, C. Y. *et al.* Succinimidyl Ester Surface Chemistry: Implications of the Competition between Aminolysis and Hydrolysis on Covalent Protein Immobilization. *Langmuir* **30**, 12868–12878 (2014).
2. Smith, J. D. *et al.* Droplet mobility on lubricant-impregnated surfaces. *Soft Matter* **9**, 1772–1780 (2013).
3. Howell, C., Grinthal, A., Sunny, S., Aizenberg, M. & Aizenberg, J. Designing Liquid-Infused Surfaces for Medical Applications: A Review. *Adv. Mater.* **30**, 1802724 (2018).
4. Zdziennicka, A., Szymczyk, K., Jańczuk, B., Longwic, R. & Sander, P. Surface, Volumetric, and Wetting Properties of Oleic, Linoleic, and Linolenic Acids with Regards to Application of Canola Oil in Diesel Engines. *Applied Sciences* **9**, 3445 (2019).
5. Walls, D. J., Meiburg, E. & Fuller, G. G. The shape evolution of liquid droplets in miscible environments. *Journal of Fluid Mechanics* **852**, 422–452 (2018).
6. Boreyko, J. B., Polizos, G., Datskos, P. G., Sarles, S. A. & Collier, C. P. Air-stable droplet interface bilayers on oil-infused surfaces. *PNAS* **111**, 7588–7593 (2014).
7. Yuk, H. *et al.* Rapid and coagulation-independent haemostatic sealing by a paste inspired by barnacle glue. *Nat Biomed Eng* **5**, 1131–1142 (2021).
