# Supplementary material for: A 3D printable tissue adhesive
Source: Nat Commun. 2024 Feb 9;15:1215. doi: 10.1038/s41467-024-45147-9 (PMC10853267; doi:10.1038/s41467-024-45147-9)
Supplement: Supplementary file 3 — Description of Additional Supplementary Files [file 41467_2024_45147_MOESM3_ESM.pdf]

Description of Additional Supplementary Files

Supplementary Movies

Supplementary Movie S1

Description: Sealing of an in vivo rat liver using the liquid-infused 3D printed patch

Supplementary Movie S2

Description: Sealing of an in vivo rat femoral artery using the liquid-infused 3D printed patch
