# Supplementary material for: A 3D printable tissue adhesive
Source: Nat Commun. 2024 Feb 9;15:1215. doi: 10.1038/s41467-024-45147-9 (PMC10853267; doi:10.1038/s41467-024-45147-9)
Supplement: Supplementary file 6 — Reporting Summary [file 41467_2024_45147_MOESM6_ESM.pdf]

nature portfolio

Corresponding author(s): Hyunwoo Yuk
Xuanhe Zhao

Last updated by author(s): Dec 7, 2023

Reporting Summary

Nature Portfolio wishes to improve the reproducibility of the work that we publish. This form provides structure for consistency and transparency in reporting. For further information on Nature Portfolio policies, see our Editorial Policies and the Editorial Policy Checklist.

Statistics

For all statistical analyses, confirm that the following items are present in the figure legend, table legend, main text, or Methods section.

n/a	Confirmed	
☐	☒	The exact sample size (*n*) for each experimental group/condition, given as a discrete number and unit of measurement
☐	☒	A statement on whether measurements were taken from distinct samples or whether the same sample was measured repeatedly
☐	☒	The statistical test(s) used AND whether they are one- or two-sided<br>*Only common tests should be described solely by name; describe more complex techniques in the Methods section.*
☒	☐	A description of all covariates tested
☐	☒	A description of any assumptions or corrections, such as tests of normality and adjustment for multiple comparisons
☐	☒	A full description of the statistical parameters including central tendency (e.g. means) or other basic estimates (e.g. regression coefficient) AND variation (e.g. standard deviation) or associated estimates of uncertainty (e.g. confidence intervals)
☐	☒	For null hypothesis testing, the test statistic (e.g. *F*, *t*, *r*) with confidence intervals, effect sizes, degrees of freedom and *P* value noted<br>*Give P values as exact values whenever suitable.*
☒	☐	For Bayesian analysis, information on the choice of priors and Markov chain Monte Carlo settings
☒	☐	For hierarchical and complex designs, identification of the appropriate level for tests and full reporting of outcomes
☒	☐	Estimates of effect sizes (e.g. Cohen's *d*, Pearson's *r*), indicating how they were calculated

Our web collection on statistics for biologists contains articles on many of the points above.

Software and code

Policy information about availability of computer code

Data collection	OMEGA PC software for USBH pressure transducers was used to collect burst pressure measurements. testXpert Testing Software was used to collect force data for tensile tests, T-peel tests, and shear strength tests. TA Rheology Advantage was used to collect data for rheological characterizations.
Data analysis	Aperio ImageScope (version 12.4.6) was used to view histological images. ImageJ (version 2.1.0) was used to analyze microscope and MicroCT images. Statistical analyses were performed using MATLAB (version 2021b) and Microsoft Excel (version 2021).

For manuscripts utilizing custom algorithms or software that are central to the research but not yet described in published literature, software must be made available to editors and reviewers. We strongly encourage code deposition in a community repository (e.g. GitHub). See the Nature Portfolio guidelines for submitting code & software for further information.

Data

Policy information about availability of data

All manuscripts must include a data availability statement. This statement should provide the following information, where applicable:

- Accession codes, unique identifiers, or web links for publicly available datasets
- A description of any restrictions on data availability
- For clinical datasets or third party data, please ensure that the statement adheres to our policy

The data supporting the findings of this study are available within the article and its supplementary files. Any additional requests for information can be directed to, and will be fulfilled by, the corresponding authors. Source data are provided with this paper.

Research involving human participants, their data, or biological material

Policy information about studies with human participants or human data. See also policy information about sex, gender (identity/presentation), and sexual orientation and race, ethnicity and racism.

Reporting on sex and gender	N/A
Reporting on race, ethnicity, or other socially relevant groupings	N/A
Population characteristics	N/A
Recruitment	N/A
Ethics oversight	N/A

Note that full information on the approval of the study protocol must also be provided in the manuscript.

Field-specific reporting

Please select the one below that is the best fit for your research. If you are not sure, read the appropriate sections before making your selection.

☒ Life sciences ☐ Behavioural & social sciences ☐ Ecological, evolutionary & environmental sciences

For a reference copy of the document with all sections, see nature.com/documents/nr-reporting-summary-flat.pdf

Life sciences study design

All studies must disclose on these points even when the disclosure is negative.

Sample size	For the in vivo rat studies, we chose a sample size of n = 3 independent samples per study group for the survival studies (i.e., subcutaneous implantation, trachea defect repair, and colon defect repair) and a sample size of n = 2 independent samples for the terminal studies (i.e., liver and femoral artery hemostasis). These sample sizes were based on previously reported works, including refs. 9, 12, 13 in the manuscript. The justification behind the small sample size is that these studies are exploratory in nature and designed to serve as a preliminary investigation into the in vivo performance of the material, rather than to draw definitive conclusions. For other (non in vivo) experiments, sample sizes were not predetermined using a statistical method. Statistical significance and p values were computed to assess whether the sample sizes yielded statistically significant results.
Data exclusions	No data were excluded.
Replication	The in vivo studies were replicated at least 3 times per study group for survival studies, and at least 2 times per study group for terminal studies. All attempts at replication were successful.
Randomization	All studies were performed with randomly allocated experimental groups.
Blinding	Blinding was not possible for the in vivo histological analyses, either due to the visible difference between experimental study groups (for studies comparing TachoSil and the experimental material) or because blinding was not relevant (for studies that did not involve a comparison between materials).

Reporting for specific materials, systems and methods

We require information from authors about some types of materials, experimental systems and methods used in many studies. Here, indicate whether each material, system or method listed is relevant to your study. If you are not sure if a list item applies to your research, read the appropriate section before selecting a response.

Materials & experimental systems

n/a	Involved in the study	
☒	☐	Antibodies
☐	☒	Eukaryotic cell lines
☒	☐	Palaeontology and archaeology
☐	☒	Animals and other organisms
☒	☐	Clinical data
☒	☐	Dual use research of concern
☒	☐	Plants

Methods

n/a	Involved in the study	
☒	☐	ChIP-seq
☒	☐	Flow cytometry
☒	☐	MRI-based neuroimaging

Eukaryotic cell lines

Policy information about cell lines and Sex and Gender in Research

Cell line source(s)	BALB/3T3 clone A31 fibroblast (American Type Culture Collection®; CCL163™).
Authentication	BALB/3T3 cells were authenticated with karyotyping by ATCC.
Mycoplasma contamination	Tested and found negative for mycoplasma contamination by ATCC.
Commonly misidentified lines (See ICLAC register)	N/A

Animals and other research organisms

Policy information about studies involving animals; ARRIVE guidelines recommended for reporting animal research, and Sex and Gender in Research

Laboratory animals	Female Sprague Dawley rats (12 weeks, 225-275 g weight) were purchased from Charles River Laboratories.
Wild animals	This study does not involve wild animals.
Reporting on sex	Female rats were used.
Field-collected samples	This study does not involve field-collected samples.
Ethics oversight	Animal procedures were reviewed and approved by the Massachusetts Institute of Technology Committee on Animal Care (MIT CAC).

Note that full information on the approval of the study protocol must also be provided in the manuscript.

Plants

Seed stocks	N/A
Novel plant genotypes	N/A
Authentication	N/A
